# Supplementary material for: An Active-Learning Resuscitation Leadership Curriculum for Emergency Medicine Residents
Source: MedEdPORTAL. 2026 Jun 17;22:11610. doi: 10.15766/mep_2374-8265.11610 (PMC13272583; doi:10.15766/mep_2374-8265.11610)
Supplement: Supplementary file 1 — Resuscitation Leaders Role.docxTeam and Situational Management.docxResuscitation Guidelines and Psychological Safety.docxResuscitation Leaders Role Review.pptxTeam and Situational Management Review.pptxResuscitation Leadership Escape Room.docxFacilitator Overview Guide.docxLBDQ Form.docxPre- and Postsurvey.docx [file mep_2374-8265.11610-s001.zip › A. Resuscitation Leaders Role.docx]

**By the end of this lecture, residents will be able to:**

1. Communicate clear expectations and roles to members of the resuscitation team during emergency department resuscitations.

**0:00 - 0:05** Brief introductions and leadership definitions

**0:05 – 0:07** Teams are given group stickers, handouts and break into groups based on leadership identity

**0:07 - 0:17** Case 1: Applying your leadership strengths

**0:17 - 0:27** Case 2: Approaching your leadership weaknesses

**0:27 - 0:30** Final discussion

**Materials Needed**

- Handouts (1 per student and facilitator)
- Discussion guides (1 per facilitator)
- Nametag Stickers (to label leadership identity)
- Pens/Markers

**People Needed**

- Lead facilitator (1)
- Small group facilitators (4-6, lead facilitator may serve as an alternate as needed)

**Lead Facilitator Instructions**

- Prior to the session, ensure all small-group facilitators have the required materials and are familiar with the session objectives and discussion structure.
- Assign each facilitator to a leadership style group (coercive, authoritative, affiliative, democratic, pacesetting, coaching). If facilitator numbers are limited, combine complementary leadership styles (e.g., coercive with pacesetting, affiliative with democratic, authoritative with coaching).
- At the start of the session, provide a brief overview of leadership styles (Appendix D). Learners should then self-identify a primary leadership style based on either pre-reading or this introductory review. No formal inventory is required. Learners should write their leadership identity on the nametag stickers.
- Instruct learners to join the group corresponding to their selected leadership style. If certain groups are underrepresented (commonly coercive or pacesetting), facilitators may either (1) redistribute learners, (2) join another group to support discussion, or (3) divide larger groups to maintain balanced group sizes.
- Ideal group size is 4–6 learners. Groups may be mixed across PGY levels to promote peer learning and diverse perspectives.
- The handout should be dispersed to learners at the end of the session.

**Pre-reading**

- Yun S, Faraj S, Sims HP. Contingent leadership and effectiveness of trauma resuscitation teams. *J Appl Psychol*. 2005;90(6):1288-1296. doi:10.1037/0021-9010.90.6.1288
- Goleman D. Leadership That Gets Results. *Harv Bus Rev*. Published 2000:78-90.
  - Optional:
    - Reid C. Making Things Happen - The Art of Leading Resuscitation. 2023. Accessed July 4, 2024. https://www.youtube.com/watch?v=Qi-TxP-Uhxg

Discussion Guide

**Instructions for facilitators**: Today you will be discussing leadership definitions, experiences, and applications in medical resuscitation scenarios through a case-based small group discussion. A brief review of the leadership styles will be provider prior to small group breakout to ensure all residents can identify a leadership style regardless of pre-session preparation. You will be assigned one of the leadership identities for the learners to join your group. There will be three case prompts and small group discussions. The flow for the cases is as below.

1. Present and discuss case part 1 in your small group (ten minutes)
2. Present and discuss case part 2 in your small group (ten minutes)
3. Reconvene with the larger group (three minutes)

Your role is to read the cases aloud to the learners, encourage every learner to participate, and participate in the discussions yourself. Specific definitions for leadership terms are included in the lecture handout at the back of this guide. For information of managing small group discussions, please refer to the curriculum facilitator overview guide (Appendix G). Facilitators should actively manage group dynamics, encouraging participation from all learners and redirecting dominant participants as needed. If group sizes are small or uneven (e.g., only one learner selects a leadership style), the lead facilitator may combine groups or reassign learners to ensure meaningful discussion.

Case part 1

Facilitator should read the following case:

*The setting is a level II trauma center in Ypsilanti, MI in a 20-bed ED. You are a single coverage physician overseeing one APP, a team of 4 nurses, one medical technician, and one respiratory therapist. If asked, a pharmacist is in house but they have to be called to the ER. You are alerted by nursing of an incoming ambulance with a critically ill patient.*

*“Hi, this is EMS with a critically ill patient. We are bringing in a 63-year-old male for cardiac arrest. The initial rhythm was ventricular fibrillation. We administered 1 mg of epinephrine and gave one shock before achieving ROSC. Total CPR time was 10 minutes. After loading him into the ambulance he became pulseless again. We have given 2 more rounds of epi, 1 round of amiodarone, and gave 2 more shocks but he is still pulseless. We will be at your facility in five minutes.*

*The patient arrives and he is moved over to your stretcher. With you are:*

- *3 nurses who have all been working with you for 5+ years*
- *A respiratory therapist who graduated 2 weeks ago but has worked with you previously on several shifts*
- *An APP who has worked for this ER for 25 years, however this is your first time working together.*

*Your most senior nurse is getting a second IV. Another nurse is timekeeping behind you. The third nurse is standing in the corner and watching. Your respiratory therapist has gone between bagging, setting up the vent, finding the suction, setting up the suction, dropping the suction, and back to bagging. Your APP is asking if they can go finish the lac repair in room 3. Your relief has arrived and is asking you multiple questions about the department not related to the current patient. As this is all happening, you begin to daydream about a group discussion you had as a resident about how to use your leadership strengths.*

Discussion questions:

1. Do you anticipate using empowering or directive leadership during this resuscitation?
2. Do you have any examples of physicians using empowering or directive leadership in a resuscitation?
3. **The facilitator reads the Goleman leadership description for your specified leadership style.**
4. What are some of the strengths of your leadership style?
5. How would you use the selected leadership style for this resuscitation?
6. What does it mean to be a resuscitation leader?

Case part 2

(**Facilitator note:** After reading the shared case introduction, select and read the scenario corresponding to the leadership style assigned to your group. Some leadership styles are paired and share a scenario as outlined below).

*You have reorganized your team and feel everything is running smoothly. You have established an airway, have 2 working 16 g IVs, have paged the on-call cardiologist, have a great cycle of compressors, and have ensured each compressor is giving compressions at an adequate rate and depth. Despite all this, the patient has remained in VFib and it has been 45 minutes since they first collapsed. Just then, the Cardiologist arrives and says:*

1. ***Authoritative****: Thank you for your help but we are going to do things my way now. Can you go print the EKGs you’ve gotten so far and order some lidocaine, I will lead now.*
2. ***Pacesetting/Coercive****: Why have you just been following ACLS? What is your plan? My approach is do always start with a precordial thump, we need to stop and try that. how I learned this - we need to do a precordial thump so let’s try that. Can we stop compressions for a minute?*
3. ***Democratic/Affiliative****: Who are you? Listen, as the cardiologist I think I need to lead this.. Look at your team, your respiratory therapist doesn’t know what they are doing, and your nurse is pushing meds wrong. I am going to take things and get things back on track.*
4. ***Coaching****: Why did you put two IVs in? Why have you pushed Epi so much? Why didn’t you use a different antiarrhythmic? Did you get the EKG? What did the EKG say? Don’t you think this looks like an ST elevation? What were you going to do next? A vector change? I think dual sequential would be better so can we try that?*

As you think of a response, you daydream back to that discussion you had at grand rounds so many years ago.

Discussion questions:

1. What are some of the challenges/weaknesses of your selected Goleman leadership strategy?
2. How might you use your strengths to overcome some of those challenges?
3. How might you practice improving your weaknesses/challenges?
4. Looking back to this scenario, what would you say to the cardiologist to recapture your team's performance?

**Lecture Handout**

**Resuscitation Leadership-** The act of guiding a team during life-threatening medical emergencies to optimize patient outcomes

**Empowering Leadership-** A leadership style that involves leaders who consult with team members and encourage them to participate in developing and implementing objectives. In a resuscitation, an empowering leader encourages team members to actively participate in decision-making and task management, asks team members to express their opinions and ideas, and delegates responsibility and authority to team members. Empowering leadership is effective when team members are experienced or when tasks are simple.

**Directive Leadership**- A hands-on, task-oriented leadership style that involves leaders taking an active role in setting clear objectives and ensuring team members follow through. In a resuscitation, a directive leader develops and finalizes the care plan with little consultation with other team members, provides detailed instructions to team members, and expects team members to follow their instructions and carry out their care plan without discussion. Directive leadership is effective when team members are inexperienced and facing a difficult task.

**Goleman Leadership Styles**

**Coercive Leadership-**“Do what I say”. Style where the leader makes all decisions and gives orders to their team without explanations. These leaders use close and tight control combined with specific rules, roles, and expectations to lead a team. Effective during high-stakes or emergent situations with difficult or novice team members. Can quickly become overbearing and turn off team members.

**Authoritative Leadership**- “Come with me”. Style where the leader develops a vision for the team with team member buy-in to ensure the group is working toward a collective goal. Team member performance is determined by how they contribute to the team's vision, giving team members partial ownership of the goal. Authoritative leaders also give team members the independence to determine the steps they will take to achieve the team’s vision. Effective in several team situations, particularly when team members are interested in contributing to the team's success. When using this style be cautious that short-term goals are still being met.

**Affiliative Leadership- “**People come first”. Style where the leader focuses on building relationships with team members and works to make them happy. Leaders use feedback, recognition, and rewards to build team unity and morale. Effective in long-term teams and when focusing on improving team communication and mending broken trust. Be cautious of allowing poor performers to continue operating without some form of constructive feedback.

**Democratic Leadership**- “What do you think”. Style where leader empowers their team to take full part in the decision-making process. The goal is to allow team members to bring forward suggestions and ideas and to reach team consensus. Effective when the leader is unsure of the best course of plan and to build commitment from team members. Be cautious as this approach can be very slow and not feasible in emergent scenarios.

**Pacesetting Leadership**- “Do as I do, now”. Style where the leader places high expectations on team members by setting an example of the work to be done. These leaders will be hands-on with team tasks and expect other team members to perform at a similar level. Effective in short-term tasks with an experienced team. Be cautious as this approach’s lack of focus on developing camaraderie and high stakes can lead to team dissolution and burnout.

**Coaching Leadership-** “Try this”. Style where the leader nurtures individual team members by giving advice and opportunities for growth. Results in team members with high commitment to team tasks and loyalty to your leadership. Effective when team members are interested in being coached either to improve a weakness or develop new strengths. This approach is more difficult to use when team members are resistant to feedback or have little ambition. Additionally, this approach takes a large commitment from the leader to work effectively.
