# Supplementary material for: An Active-Learning Resuscitation Leadership Curriculum for Emergency Medicine Residents
Source: MedEdPORTAL. 2026 Jun 17;22:11610. doi: 10.15766/mep_2374-8265.11610 (PMC13272583; doi:10.15766/mep_2374-8265.11610)
Supplement: Supplementary file 1 — Resuscitation Leaders Role.docxTeam and Situational Management.docxResuscitation Guidelines and Psychological Safety.docxResuscitation Leaders Role Review.pptxTeam and Situational Management Review.pptxResuscitation Leadership Escape Room.docxFacilitator Overview Guide.docxLBDQ Form.docxPre- and Postsurvey.docx [file mep_2374-8265.11610-s001.zip › B. Team and Situational Management.docx]

**By the end of this lecture, residents will be able to:**

1. Direct and coordinate team actions during resuscitations by assigning tasks, prioritizing measures, and guiding how interventions should be performed.

**0:00 - 0:10** Brief introductions and review of pre-work material

**0:10 - 0:25** Case 1: Global Perspectives

**0:25 - 0:40** Case 2: Shared Mental Models

**0:40 - 0:55** Play the resuscitation space game

**0:55 - 1:00** Debrief

**Materials Needed**

- Discussion Guides (1 per facilitator)
- Handouts (1 per student and facilitator)
- Post surveys (1 per student)
- 2 Role-playing scripts per group (8-12 total)
- 3 Resus room sheets per group (12-18 total)

**Lead Facilitator Instructions**

- Prior to the session, ensure all small-group facilitators have the required materials and are familiar with the session objectives and discussion structure.
- At the start of the session, provide a brief overview of key team and situational management concepts (Appendix E). The aim of this review is to ensure residents are aware of the key team management and communication concepts that will be covered in the session.
- Learners should be broken up into 4-6 groups depending on the number of facilitators available for discussion. Ideal group size is 4–6 learners. Groups may be mixed across PGY levels to promote peer learning and diverse perspectives.
- The lead facilitator will keep time for groups and ensure groups are progressing through the cases and game appropriately. For the role-play cases, the role-play should take no more than 7 minutes. For the game, each scenario should take 5 minutes.

**Pre-reading**

- Mellick LB, Adams BD. Resuscitation Team Organization for Emergency Departments: A Conceptual Review and Discussion. *Open Emerg Med J*. 2009;2:18-27. (Read pages 21-25)
- Tschan F, Semmer NK, Hunziker S, Kolbe M, Jenni N, Marsch SU. Leadership in different resuscitation situations. *Trends Anaesth Crit Care*. 2014;4(1):32-36. doi:10.1016/j.tacc.2013.12.001 (Read pages 33-35)
- Kelleher DC, Kovler ML, Waterhouse LJ, Carter EA, Burd RS. Factors affecting team size and task performance in pediatric trauma resuscitation. *Pediatr Emerg Care*. 2014;30(4):248-253. doi:10.1097/PEC.0000000000000106,
- van Rensburg JJ, Santos CM, de Jong SB, Uitdewilligen S. The Five-Factor Perceived Shared Mental Model Scale: A Consolidation of Items Across the Contemporary Literature. *Front Psychol*. 2022;12:784200. doi:10.3389/FPSYG.2021.784200/BIBTEX (Read pages 1-6)
  - Optional:
    - American Hospital Association. Closed-Loop Communication | AHA TeamSTEPPS Video Toolkit. Accessed August 11, 2024. <https://www.aha.org/center/project-firstline/teamstepps-video-toolkit/closed-loop-communication>
    - American Hospital Association. Shared Mental Model | AHA TeamSTEPPS Video Toolkit. Accessed August 11, 2024. https://www.aha.org/center/project-firstline/teamstepps-video-toolkit/shared-mental-model

Discussion Guide

**Instructions for facilitators**: Today you will be discussing key concepts of resuscitation team management, task delineation, and team communication. This includes focusing on shared mental models, global perspectives, closed-loop communication, and the roles of resuscitation team members. The session is broken into two interactive activities: role-playing case based scenarios and a role assignment game.

*Role-playing cases:* There are two role playing cases with the goal of stressing the importance of team management and communication concepts/techniques. At the start of each case, ask for two volunteers, one to serve as the physician and one to serve as the nurse. Any resident may volunteer, though we encourage more junior residents to be involved.

Once the volunteers are identified you will hand them their script. These scripts explain their role, what information they are aware of, and that they should not share the written information during the role-play unless very specifically prompted (ex: the doctor asks for all vital signs). Instruct the two volunteers to work together, with their given limitations, to the conclusion of the case.

These cases are structured in a way to show what happens when teams do not use team management and communication techniques (shared a mental model, global perspective, closed-loop communication). Cases will either end with the patient being properly treated or a poor outcome occurring, with the latter designed to happen more frequently. If learners rapidly identify key issues and move toward appropriate management early, facilitators may allow the case to progress successfully and should shift discussion toward what behaviors enabled success.

You should allow up to 7 minutes of role-playing before moving on to the discussion. The lead facilitator will give you prompts to transition to the discussion. If discussion becomes overly focused on diagnosis or clinical management, facilitators should redirect learners to reflect on communication, leadership behaviors, and team coordination. At the end of each case, ask for general thoughts from the role-play characters and audience on how they thought the case flowed and how using the team management and communication techniques may have improved the patient’s care. Discussion questions are included at the end of the cases to review how to us closed-loop communications, global perspectives, and shared mental models.

*Resuscitation room game*: In the final activity, you will lead the learners in a resuscitation room game with the aim of delineating roles and spaces within the resuscitation room for the specific individuals to occupy. There are three total cases to run through with your team. You will read case scenarios which include placing certain medical staff within the resuscitation room. The group should work together to identify where these individuals should be placed on the provided resuscitation room template and what responsibilities they have. **Resuscitation room sheets should be distributed to learners immediately prior to this activity. These tools are introduced during this session and do not require prior exposure.** Discussion should focus on resuscitation roles, responsibilities, and utilizing the resuscitation room environment.

**Case 1**

| **Nurse**: DO NOT SHARE THIS INFORMATION UNLESS SPECIFICALLY ASKED DURING THE ROLE-PLAY. While you are resuscitating the patient you notice that the blood pressure in the right arm is 200/120 but it is 90/50 in the left arm. When you take the EKG you notice that QRS height goes up and down each beat. | **Doctor**: DO NOT SHARE THIS INFORMATION UNLESS SPECIFICALLY ASKED DURING THE ROLE-PLAY. While waiting for results you conduct a chart review. You find that the patient has a history of an ischemic stroke 10 years prior, has a history of poorly controlled hypertension, and uses tobacco. They are not on blood thinners. The initial head CT you ordered shows no evidence of intracranial pathology. |
| --- | --- |

**Case**: John a 72-year-old male arrives by ambulance with a chief complaint of difficulty speaking and walking. Family said he woke up normally and talking and when they checked on him after 30 minutes he was stumbling and slurring his words. It does not appear as though he fell. The family does not know any other preceding history. On your initial exam, the patient is dysarthric with L-sided facial droop and left lower extremity paralysis. His vitals are 200/120, HR 124, RR 24, T 36.4 C, and O2 of 97% on RA. The patient is now back from a CT scan. Begin your resuscitation.

***Case flow****-*

- *If asked for* ***exam findings****, the patient is tachycardic, dysarthric with L-sided facial droop and LLE paralysis. His exam is otherwise unremarkable.*
- *If asked about* ***contraindications to tPA/TNK*** *state there are none.*
- *If the team* ***orders an echo*** *state that a moderate pericardial effusion with a visualized dissection flap is present.*
- *If the patient* ***receives tPA/TNK*** *state that the patient goes into cardiac arrest and the case ends.*
- *If* ***cardiothoracic surgery is consulted****, the patient is taken to the OR and the case ends.*

Discussion (ask for both the role-players and larger audience’s perspectives):

- What happened in this case?
- What went well from a communication perspective?
- What did not go well?
- What made this case difficult?
- How would maintaining a global perspective help in this case?
- How would sharing a mental model help in this case?
- If you could redo this case, what would you do differently and why?

**Case 2**

| **Nurse**: DO NOT SHARE THIS INFORMATION UNLESS SPECIFICALLY ASKED DURING THE ROLE-PLAY. You are taking over for another nurse who went on break. The other nurse told you that the patient probably has sepsis and will need a lumbar puncture. Only 1 IV is in place and you are waiting for an US IV team member to place another. In the meantime, you are only giving fluids. You have not started antibiotics. You get busy with another patient while the doctor is gone so you do not know if your patient went to CT. | **Doctor**: DO NOT SHARE THIS INFORMATION UNLESS SPECIFICALLY ASKED DURING THE ROLE-PLAY. When going to CT you ask the nurse to tell you if they see anything wrong otherwise you will proceed with the lumbar puncture. The other patient who coded took you away for 45 minutes. |
| --- | --- |

**Case**: A 45 yo male with a history of HIV presents with fever and confusion. Family tells you he has been complaining of headache and neck pain for the past three days and was making no sense this morning. He does not consistently take his HIV medicines and his family does not know what a CD4 is. He is altered but protecting his airway. He has a positive Kernig and Brudzinski sign. On chart review, you see a history of strep meningitis 2 years prior. His vitals are BP 87/50, HR 125, RR 22, T 39.2 C, O2 95% on 2 L. As you are beginning the resuscitation a patient receiving CPR comes in and the doctor has to step out into the other room. The resuscitation resident said they would put in some orders and send the patient to the CT scanner. The code has ended and the doctor is back. Continue the resuscitation.

***Case Flow****:*

- *If the patient* ***receives fluids****, their BP and HR improve.*
- *If the patient* ***doesn’t receive antibiotics****, the patient's BP and HR will gradually worsen requiring pressors.*
- *If the patient continues to* ***not receive antibiotics after pressors*** *are initiated, they will demonstrate progressive hemodynamic instability before progressing to cardiac arrest and the case ends.*
- *If the patient receives a* ***lumbar puncture****, the patient will go into cardiac arrest and the case ends*
- *If a* ***CT scan*** *is actually done a large intracranial mass will be detected.*
- *If* ***neurosurgery*** *is consulted, they will go to the OR and the case*

Discussion (ask for both the role-players and larger audience’s perspectives):

- What happened in this case?
- What went well from a communication perspective?
- What did not go well?
- What made this case difficult?
- How would closed-loop communication have helped in this case?
- How would a shared mental model have helped in this case?
- If you could redo this case, what would you do differently and why?

**Activity 3**

**Facilitator**: Read the following to the group- “For this activity, you will practice deciding roles and responsibilities during a resuscitation. We have a layout of our resuscitation rooms with spaces where team members can be placed. For each case, you will be given a list of available team members. As a team, we need to come up with where team members should be placed in the room, their roles, and their responsibilities. You may place as many team members outside of the room as you would like.”

**Case 1:** “Hi this is the fire department coming in with a critically ill patient. We have a 73-year-old woman in cardiac arrest. We initially had ROSC on scene but just lost pulses and have restarted CPR. We have a L tibial IO as our only access. We also placed an LMA. We will be at your facility in ten minutes.”

1. There is 1 attending, 1 PGY 3s, 1 PGY2s, 2 PGY1s, 2 nurses, 2 technicians, and 1 respiratory therapist.
   1. Who are we putting where? Why?
   2. What role should they have? Why?
   3. What responsibilities will they have? Why?

**Case 2**: “This is EMS bringing in a 27-year-old in active labor. She is fully dilated and told us she feels the need to push. She has a history of two other pregnancies needing C-sections due to fetal distress. We will be at your facility in five minutes.”

1. There is 1 attending, 1 PGY 1, 3 nurses, 1 technician, 1 respiratory therapist, and 3 M4 medical students.
   1. Who are we putting where? Why?
   2. What role should they have? Why?
   3. What responsibilities will they have? Why?

**Case 3**: Your trauma clerk tells you the local police department just called stating a 4 yo is coming in from the beach after drowning. Vitals are HR 145, RR45, SPO2 89% on nonrebreather, T 35.8 C, and BP 90/50. GCS is 7. The officer said the patient was coming by EMS and mom and dad were in the ambulance with the patient.

1. There is 1 PEM attending, 1 EM attending, 1 PGY3, 2 PGY2s, 3 PGY1s, 1 PGY2 pediatrics rotator, 2 nurses, 2 technicians, 2 M4 medical students, 1 respiratory therapist, 2 pharmacists, the mother, the father, and a chaplain.
   1. Who are we putting where? Why?
   2. What role should they have? Why?
   3. What responsibilities will they have? Why?


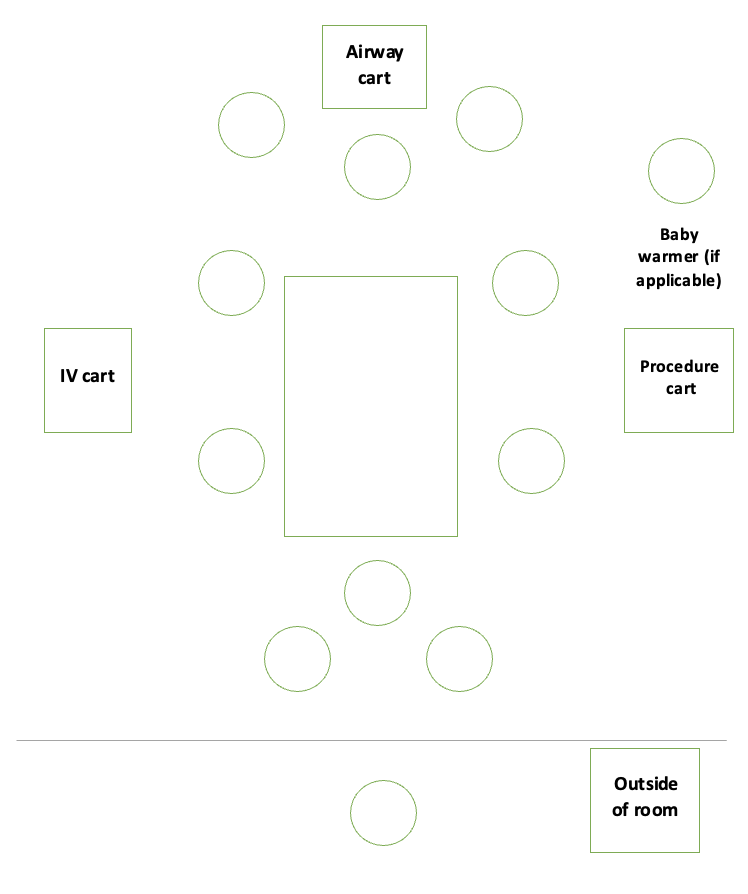


Author Owned

**Lecture Handout**

**Shared mental model-** a common understanding and internal representation of knowledge among team members. This enhances team coordination, prediction of behaviors, and adaptation to changing demands in collaborative tasks. Overall, a shared mental model gives the group a 360-degree view of the resuscitation. There are five key dimensions of the shared mental model which together allow team members to understand *which* tools or equipment to use, *what* tasks to perform, *with whom* they need to interact and coordinate, *how* to do so, and *when* the work has to be accomplished. These five dimensions include:

- Equipment (tools and technology)
- Execution (procedures, strategies, and contingency plans)
- Composition (team members’ preferences, skills, and habits)
- Temporal (duration of resuscitation, duration of procedural efforts)
- Interaction (team members’ responsibilities and communication patterns)

**Global Perspective-** an open mindset of resuscitation team leader which facilitates effective tracking of all dynamics and actions during a resuscitation. In practice, this appears as the team leader being aware of actions taken around them and team members paying attention to the flow of the resuscitation. This allows the team to avoid missing actions or making mistakes and allows for a shared mental model to be developed.

**Closed-loop communication-** a communication framework where verbal feedback ensures messages are correctly understood by recipients. In closed-loop communication, the person receiving instruction or information repeats it back to ensure the message is understood correctly and to confirm to the sender that the information is heard. This tool helps protect patients from communication errors that can lead to serious consequences.
