# Supplementary material for: An Active-Learning Resuscitation Leadership Curriculum for Emergency Medicine Residents
Source: MedEdPORTAL. 2026 Jun 17;22:11610. doi: 10.15766/mep_2374-8265.11610 (PMC13272583; doi:10.15766/mep_2374-8265.11610)
Supplement: Supplementary file 1 — Resuscitation Leaders Role.docxTeam and Situational Management.docxResuscitation Guidelines and Psychological Safety.docxResuscitation Leaders Role Review.pptxTeam and Situational Management Review.pptxResuscitation Leadership Escape Room.docxFacilitator Overview Guide.docxLBDQ Form.docxPre- and Postsurvey.docx [file mep_2374-8265.11610-s001.zip › C. Resuscitation Guidelines and Psychological Safety.docx]

By the end of this lecture, residents will be able to:

1. Apply standardized resuscitation guidelines to guide team decision-making.
2. Maintain clear performance expectations during the resuscitation.
3. Demonstrate leadership behaviors that promote a composed, supportive, and psychologically safe team environment during the resuscitation.

**0:00** Teams are given handouts and break into small groups

**0:01 - 0:20** Case part 1- Briefing and psychological safety

**0:21 - 0:40** Case part 2- Ending a resuscitation and debriefing

**0:40 - 0:45** Closing Thoughts

**Materials Needed**

- Handouts (1 per student and facilitator)
- Discussion guides (1 per facilitator)

**People Needed**

- Lead facilitator (1)
- Small group facilitators (4-6, lead facilitator may serve as an alternate as needed)

**Lead Facilitator Instructions**

- Prior to the session, ensure all small-group facilitators have the required materials and are familiar with the session objectives and discussion structure.
- At the start of the session, separate learners into small groups. Learners should be broken up into 4-6 groups depending on the number of facilitators available for discussion. Ideal group size is 4–6 learners. Groups may be mixed across PGY levels to promote peer learning and diverse perspectives.
- The lead facilitator will keep time for groups and ensure groups are progressing through the cases.
- Given the emotionally challenging nature of these cases, facilitators should monitor for learner distress and normalize emotional responses. If needed, facilitators may pause the discussion, offer brief reflection, or check in with individual learners. Additional processing can occur during the final debrief.

**Pre-reading**

- Reid C, Brindley P, Hicks C, et al. Zero point survey: a multidisciplinary idea to STEP UP resuscitation effectiveness. *Clin Exp Emerg Med*. 2018;5(3):139-143. doi:10.15441/ceem.17.269
- Lauria MJ, Gallo IA, Col Stephen Rush L, Brooks J, Spiegel R, Weingart SD. Psychological Skills to Improve Emergency Care Providers’ Performance Under Stress. *Ann Emerg Med*. Published online 2017:1-7. doi:10.1016/j.annemergmed.2017.03.018
- Torke AM, Bledsoe P, Wocial LD, Bosslet GT, Helft PR. CEASE: A Guide for Clinicians on How to Stop Resuscitation Efforts. *Ann Am Thorac Soc*. 2015;12(3):440-445. doi:10.1513/AnnalsATS.201412-552PS
- Kessler DO, Cheng A, Mullan PC. Debriefing in the emergency department after clinical events: A practical guide. *Ann Emerg Med*. 2015;65(6):690-698. doi:10.1016/j.annemergmed.2014.10.019
  - Optional:
    - Reid C. When Should Resuscitation Stop. 2014. Accessed August 25, 2024. <https://www.youtube.com/watch?v=kJgRs3rsjdE>
    - EM:RAP. Annals of Emergency Medicine: Post Resuscitation Debriefing. September 2017. Accessed August 25, 2024. https://www.emrap.org/episode/mildlyacidotic/annalsof

Discussion Guide

**Instructions for facilitators**: Today you will discuss guidelines and algorithms for leading a resuscitation focusing on team and personal psychological safety. There will be two case prompts and small group discussions. The lead facilitator will provide timekeeping for the groups. The flow for the cases is as below.

1. Present and discuss case part 1 in your small group (twenty minutes)
2. Present and discuss case part 2 in your small group (twenty minutes)
3. Reconvene with the larger group (five minutes)

Case part 1 focuses on preparing yourself, your team, and your environment prior to a stressful patient encounter. In particular, we want discussions to focus on psychologically preparing yourself and your team for the encounter. The case will reference the zero-point survey, an organized approach to preparing teams and leaders for incoming critically ill patients. You should review the content and steps of the survey (STEP-UP: self, team, environment, patient, update, priorities) when going through the discussion questions related to team and environmental preparation. Additionally, you will discuss approaches to maintaining personal psychological safety prior to and during the encounters. Beat the stress fool is a mnemonic provided as an example approach of personal preparation for difficult cases (breath, talk, see, focus) and should be applied when discussing personal preparation.

Case part 2 focuses on ending the resuscitation and debriefing. This case will focus on identifying factors important in ending a resuscitation and the objectives and impacts of debriefing after resuscitations. The CEASE mnemonic (clinical features, effectiveness, ask, stop, explain) is provided as an example framework of ending a resuscitation and should be reviewed during the discussion. DISCERN is a debriefing tool used for emergency resuscitation reviews. You should guide learners through each DISCERN step explicitly to model a structured debrief.

Your role is to present the cases to the learners and encourage active participation of all learners. If you need to reference specific resuscitation guidelines and algorithms they are included in the handout at the back of this guide. The content of these cases can be overwhelming and evoke strong emotions from residents. While we encourage open discussion and reflection, be cognizant when discussions may be going **too far or becoming emotionally overwhelming. Facilitators should redirect discussion, summarize key points, and ensure psychological safety is maintained.** The lead facilitator will be available to pull individual learners aside if needed. Please also reference the facilitator overview guide (Appendix G) for additional small group instruction guidance.

**Case Part 1**

*This is Detroit EMS coming in with lights and sirens traffic for a drowning patient. The patient is a 27-year-old male who was working on a boat when he fell overboard. He was in the water for approximately 15 minutes. He was given initial CPR at the scene had ROSC and was speaking but incoherent. He also has wounds to his head but no other obvious injuries. His vitals are BP 98/54, HR 125, RR 28, SPO2 92% on NRB. GCS is currently 10. We have given 100 mcg of fentanyl. We will be at your facility in ten minutes.*

- Can you all think of a time you had a case like this, with an incoming patient that felt stressful or anxiety provoking? Tell us about it. What happened in those moments before and as that patient arrived?
- How do you prepare yourself for this incoming patient?
- How do you prepare your team for this incoming patient?
- How do you prepare your environment for this incoming patient?
- We talked so far about preparing ourselves, our team, and our resuscitation bay for this patient. Do you feel better about the impending resuscitation you are about to lead? If yes, why? If not, why not?
  - *Facilitator notes- feel free to refer to the concept and algorithms below*
    - ***Zero Point Survey: STEP-UP-*** *Self, Team, Environment, Patient, Update, Priorities*
    - ***Beat The Stress, Fool-*** *Breathe, Talk, See, Focus*

**Case Part 2**

*The patient has arrived and is transferred to your stretcher. You get an initial set of vitals as follows: BP 67/32, HR 141 BMP, SPO2 82% on NRB, GCS is 5. Upon exposing the patient you find that he has a significant head injury with exposed brain matter. While completing the primary survey the patient loses pulses and your team begins CPR. After resuscitating the patient for 15 minutes your charge nurse alerts you that a family member of the patient is here.*

- When should this resuscitation end?
- How would you end this resuscitation?
- Think about a similar resuscitation you have been a part of. What happened once that resuscitation ended? Do you wish anything different happened?
- Why should we debrief this resuscitation?
- What would a debriefing of this resuscitation look like?
- *If time-* What would you do if the family member who shows up is not the patient’s next of kin?
  - *Facilitator notes- feel free to refer to the concept and algorithms below*
    - ***CEASE****: Clinical features, effectiveness, ask, stop, explain*
    - ***DISCERN debriefing tool***
      - What went well during our care for the patient?
      - What could have improved during our care for the patient?
      - What are potential solutions?
      - Was the team leader the only provider calling out orders?
      - Was anyone confused about who was the team leader?

**D. Lecture Handout**

Zero-Point Survey: An organized approach to preparing yourself, briefing your team, and setting up your resuscitation environment for an incoming patient. The survey is meant to precede the primary survey before the patient arrives in your resuscitation bay. The survey uses the mnemonic STEP-UP (Self, Team, Environment, Patient, Update, Priorities).

CEASE: An algorithmic approach to deciding when and how to end resuscitative efforts that incorporates team and family into decision making steps using shared mental models. Steps include 1) Identifying **C**linical features that predict good and poor outcomes, 2) Determine how **E**ffective ongoing and potential resuscitative efforts will be, 3) **A**sk team members for input, 4) **S**top resuscitative efforts if indicated, 5) and **E**xplain to family and team what happened.

Debriefing: A facilitated or guided reflection of experiential learning. In emergency medicine, it is a powerful quality and educational tool that can help improve team behavior and patient outcomes. Several tools have been developed to assist leading a debriefing including the DISCERN (Debriefing In Situ Conversation in Emergency Room Now) tool.

Psychological Safety: The belief that one is allowed to take risks, express ideas and concerns, speak up with questions, and admit mistakes without fear. Creating this setting allows for improved cognitive and technical performance of a leader and their team. Psychological safety is threatened by excess stress and anxiety. Methods to control and re-evaluate perceived stress, called performance enhancing psychological skills, can assist in improved team performance. One such method is Beat the Stress, Fool!
