# Supplementary material for: An Active-Learning Resuscitation Leadership Curriculum for Emergency Medicine Residents
Source: MedEdPORTAL. 2026 Jun 17;22:11610. doi: 10.15766/mep_2374-8265.11610 (PMC13272583; doi:10.15766/mep_2374-8265.11610)
Supplement: Supplementary file 1 — Resuscitation Leaders Role.docxTeam and Situational Management.docxResuscitation Guidelines and Psychological Safety.docxResuscitation Leaders Role Review.pptxTeam and Situational Management Review.pptxResuscitation Leadership Escape Room.docxFacilitator Overview Guide.docxLBDQ Form.docxPre- and Postsurvey.docx [file mep_2374-8265.11610-s001.zip › D. Resuscitation Leaders Role Review.pptx]

## Slide 1
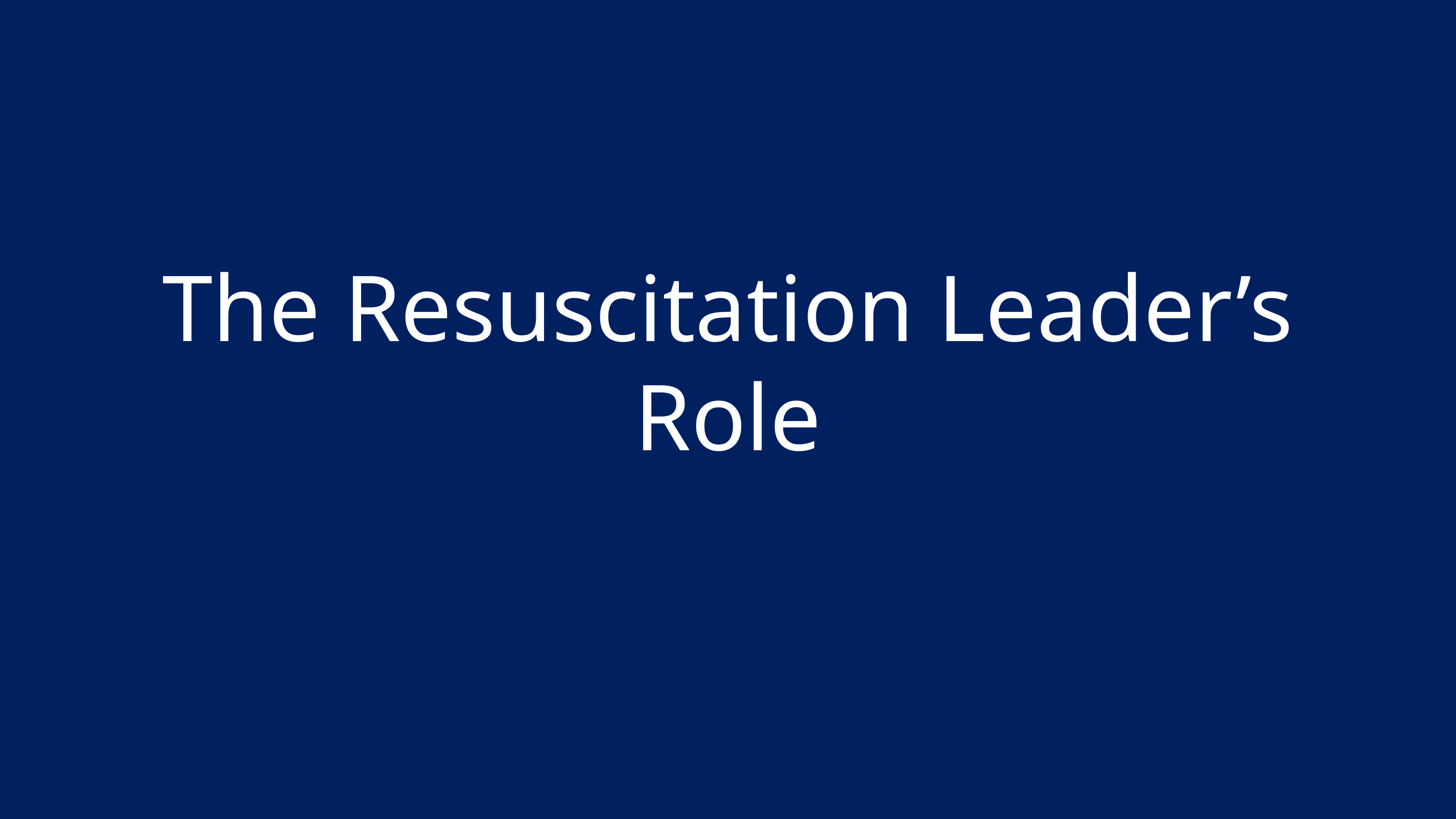

The Resuscitation Leader’s Role

## Slide 2
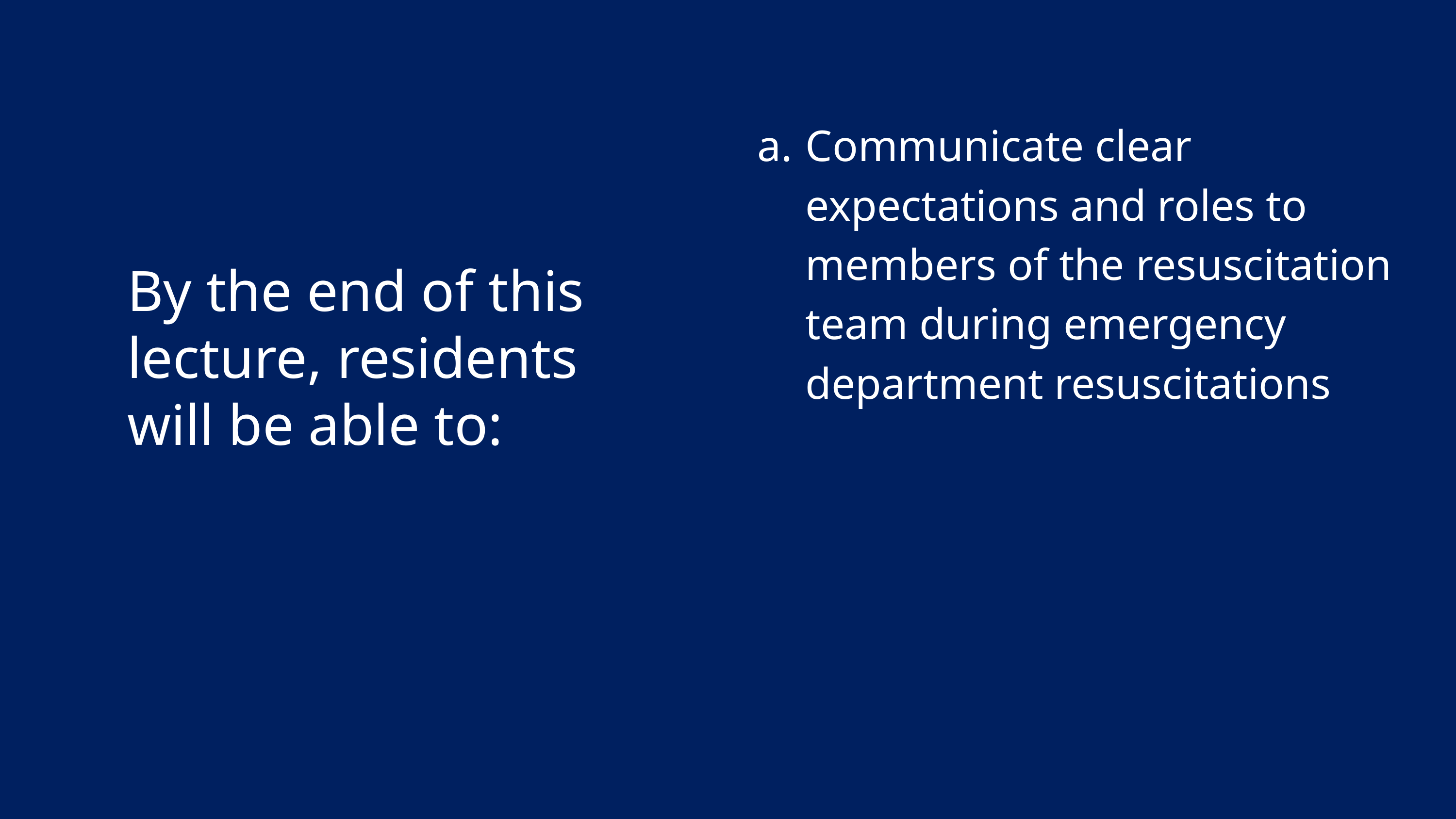

Communicate clear expectations and roles to members of the resuscitation team during emergency department resuscitations
By the end of this lecture, residents will be able to:

## Slide 3
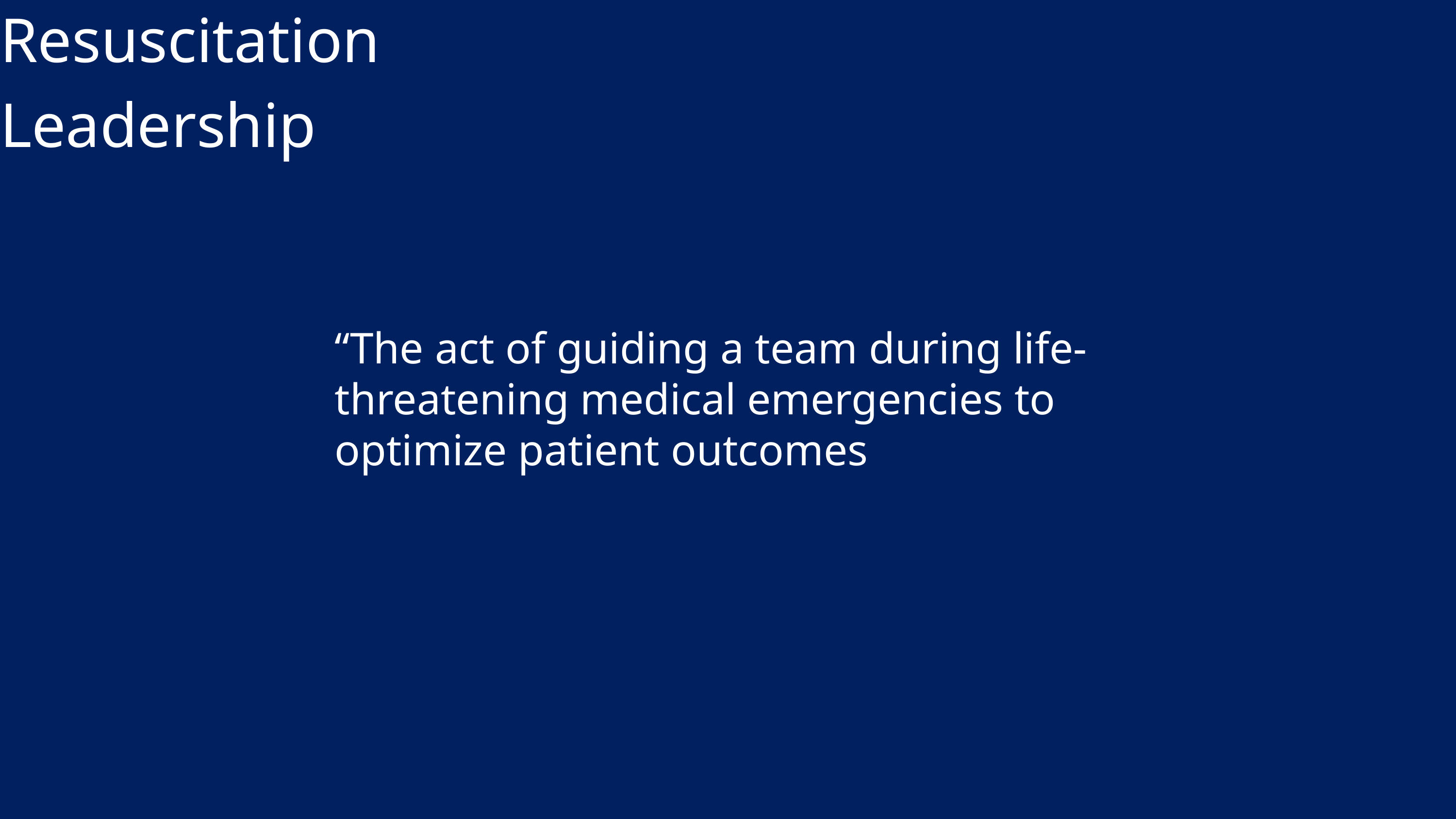

Resuscitation Leadership
“The act of guiding a team during life-threatening medical emergencies to optimize patient outcomes

## Slide 4
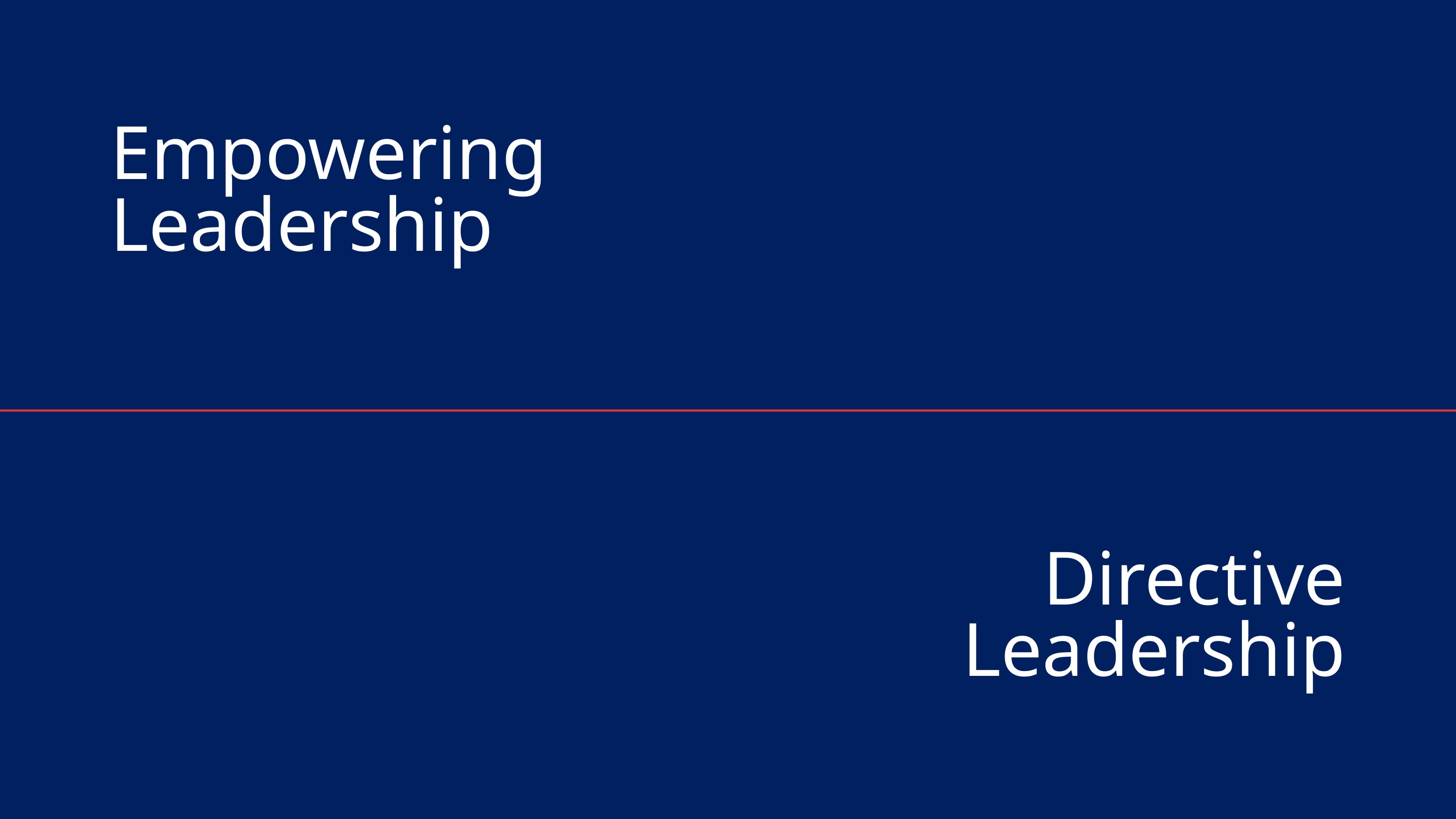

Empowering Leadership
Directive Leadership

## Slide 5
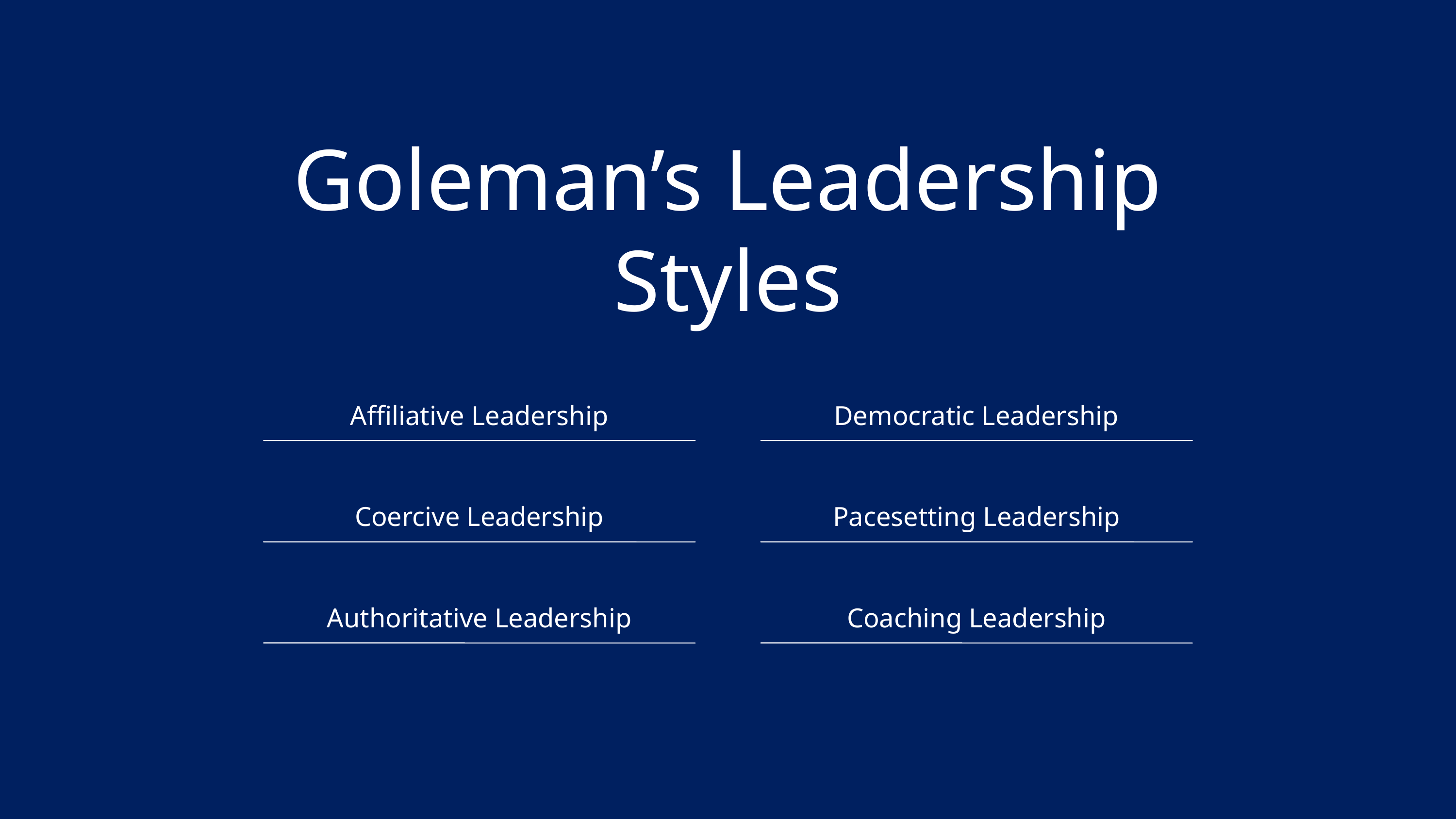

Goleman’s Leadership Styles
Affiliative Leadership
Democratic Leadership
Coercive Leadership
Pacesetting Leadership
Authoritative Leadership
Coaching Leadership

## Slide 6
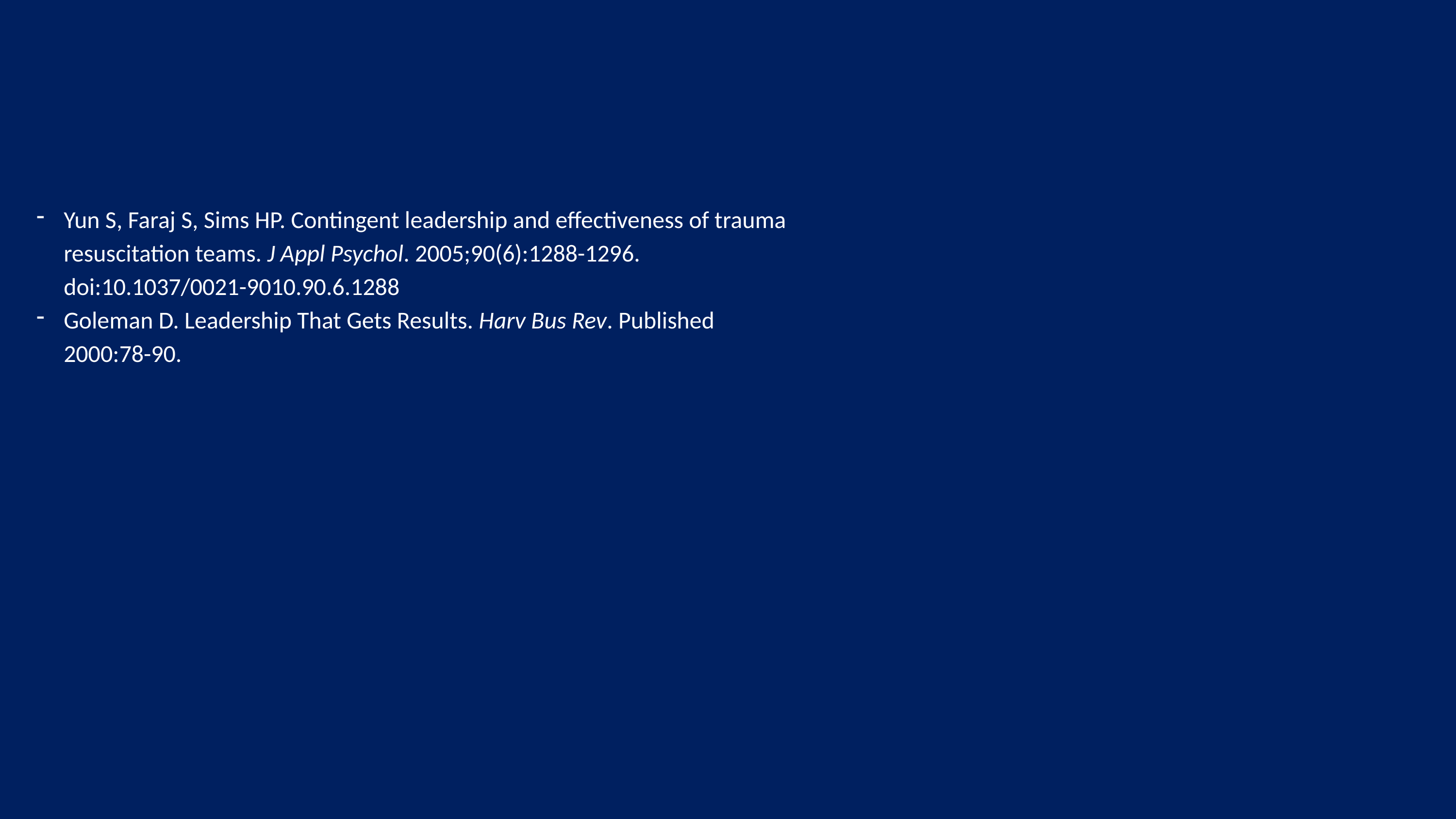

Yun S, Faraj S, Sims HP. Contingent leadership and effectiveness of trauma resuscitation teams. J Appl Psychol. 2005;90(6):1288-1296. doi:10.1037/0021-9010.90.6.1288
Goleman D. Leadership That Gets Results. Harv Bus Rev. Published 2000:78-90.
