# Supplementary material for: An Active-Learning Resuscitation Leadership Curriculum for Emergency Medicine Residents
Source: MedEdPORTAL. 2026 Jun 17;22:11610. doi: 10.15766/mep_2374-8265.11610 (PMC13272583; doi:10.15766/mep_2374-8265.11610)
Supplement: Supplementary file 1 — Resuscitation Leaders Role.docxTeam and Situational Management.docxResuscitation Guidelines and Psychological Safety.docxResuscitation Leaders Role Review.pptxTeam and Situational Management Review.pptxResuscitation Leadership Escape Room.docxFacilitator Overview Guide.docxLBDQ Form.docxPre- and Postsurvey.docx [file mep_2374-8265.11610-s001.zip › E. Team and Situational Management Review.pptx]

## Slide 1
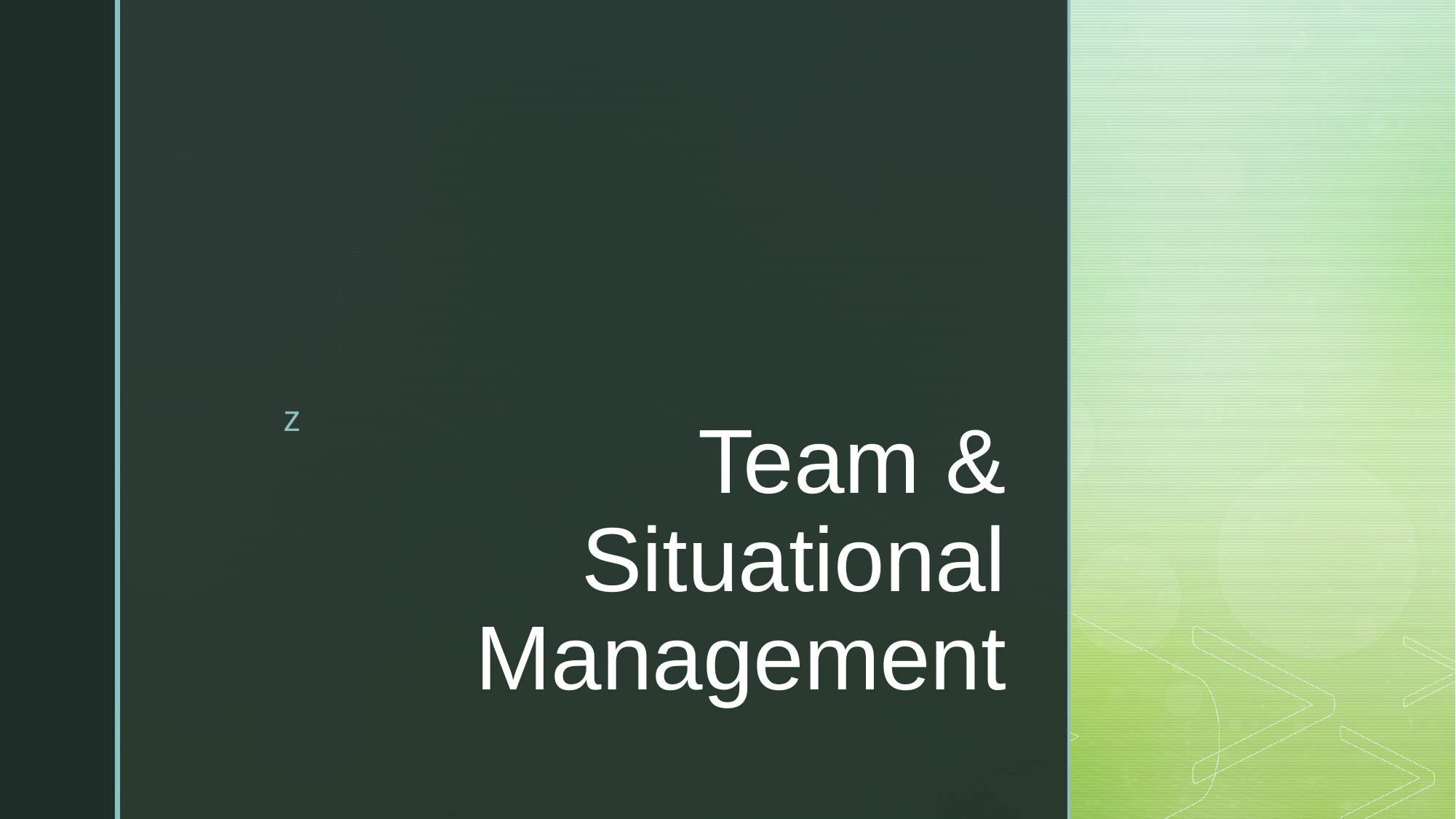

# Team & Situational Management

## Slide 2
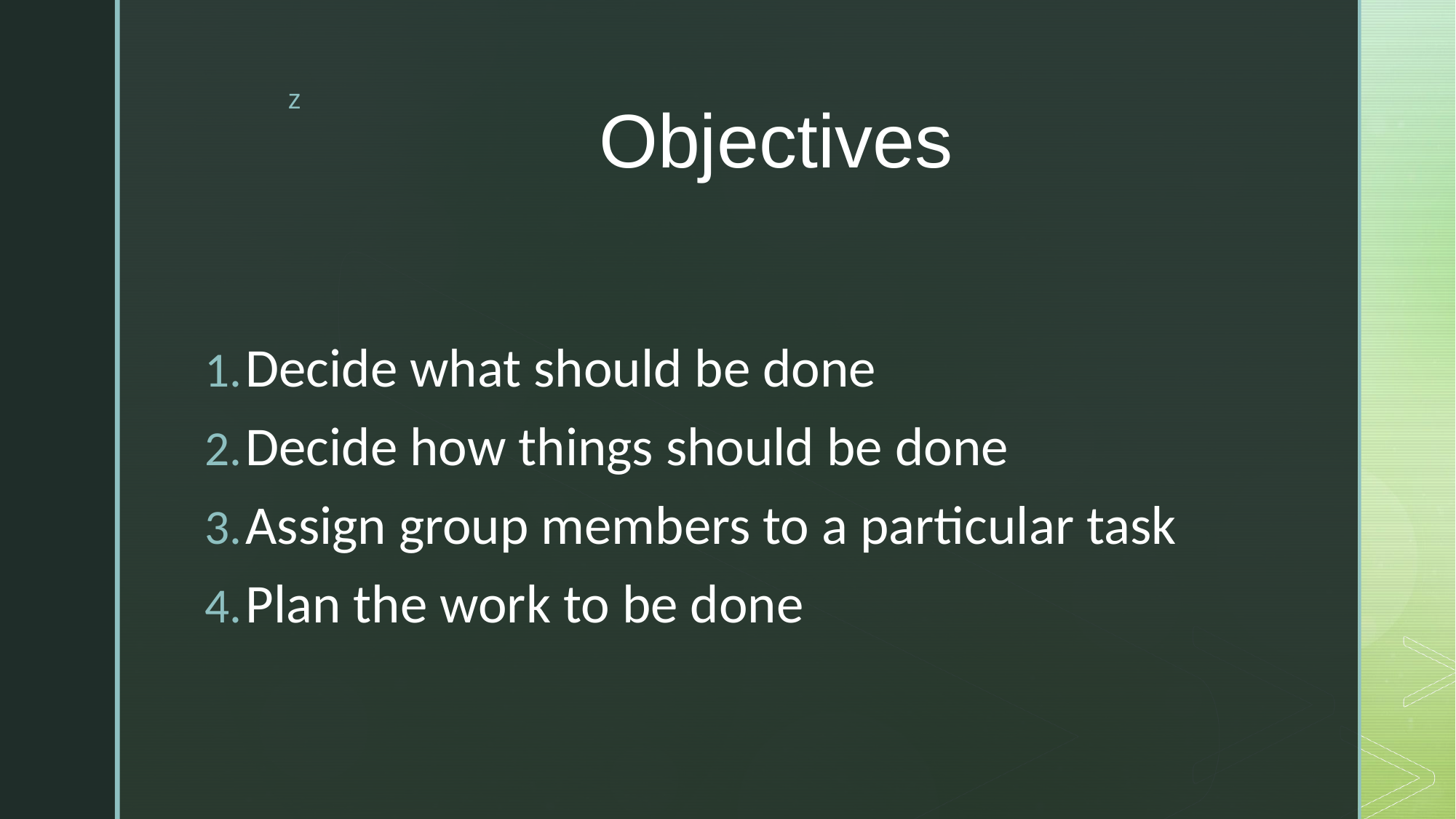

# Objectives
Decide what should be done
Decide how things should be done
Assign group members to a particular task
Plan the work to be done

## Slide 3
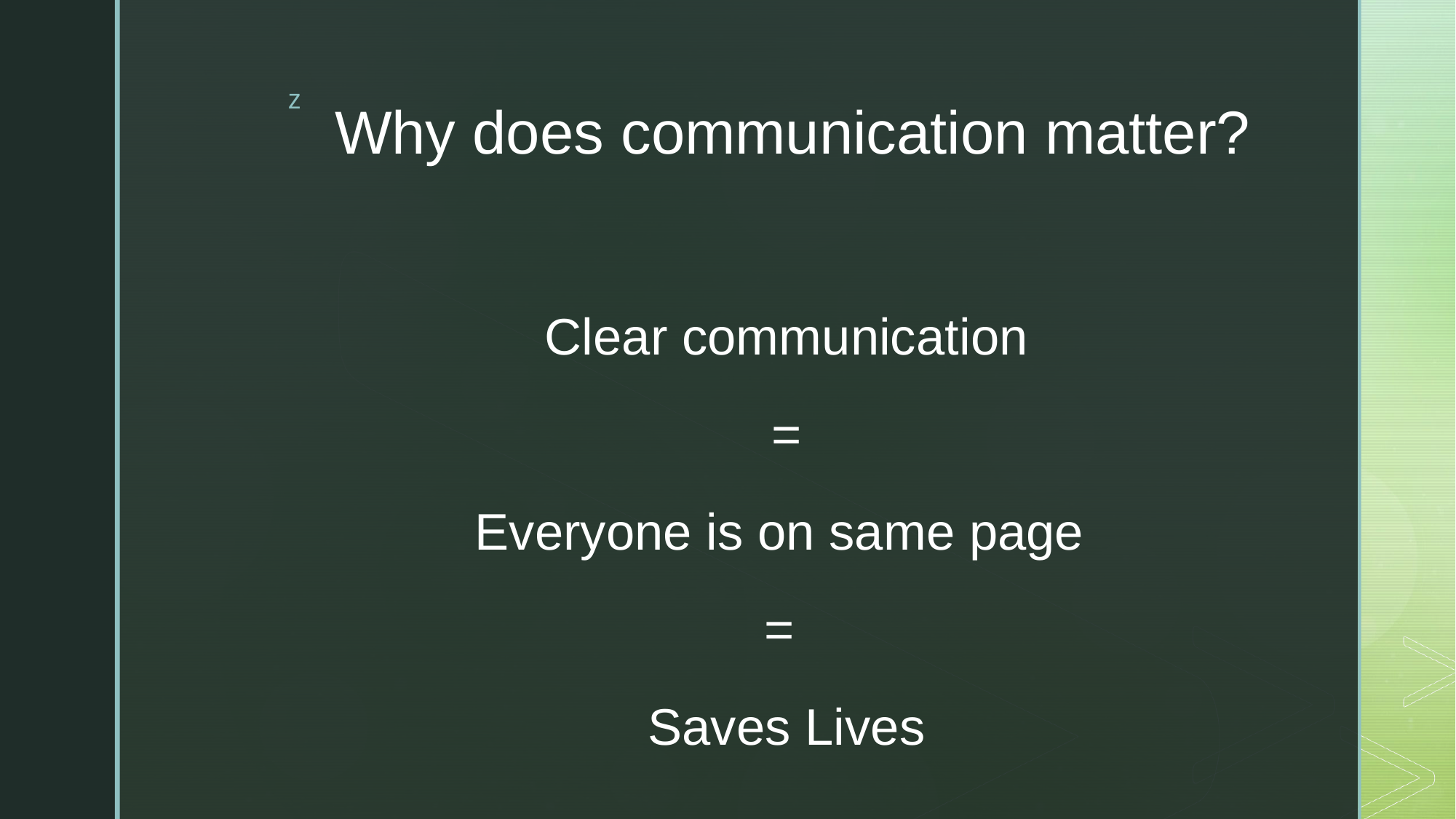

# Why does communication matter?
Clear communication
 =
Everyone is on same page
=
Saves Lives

## Slide 4
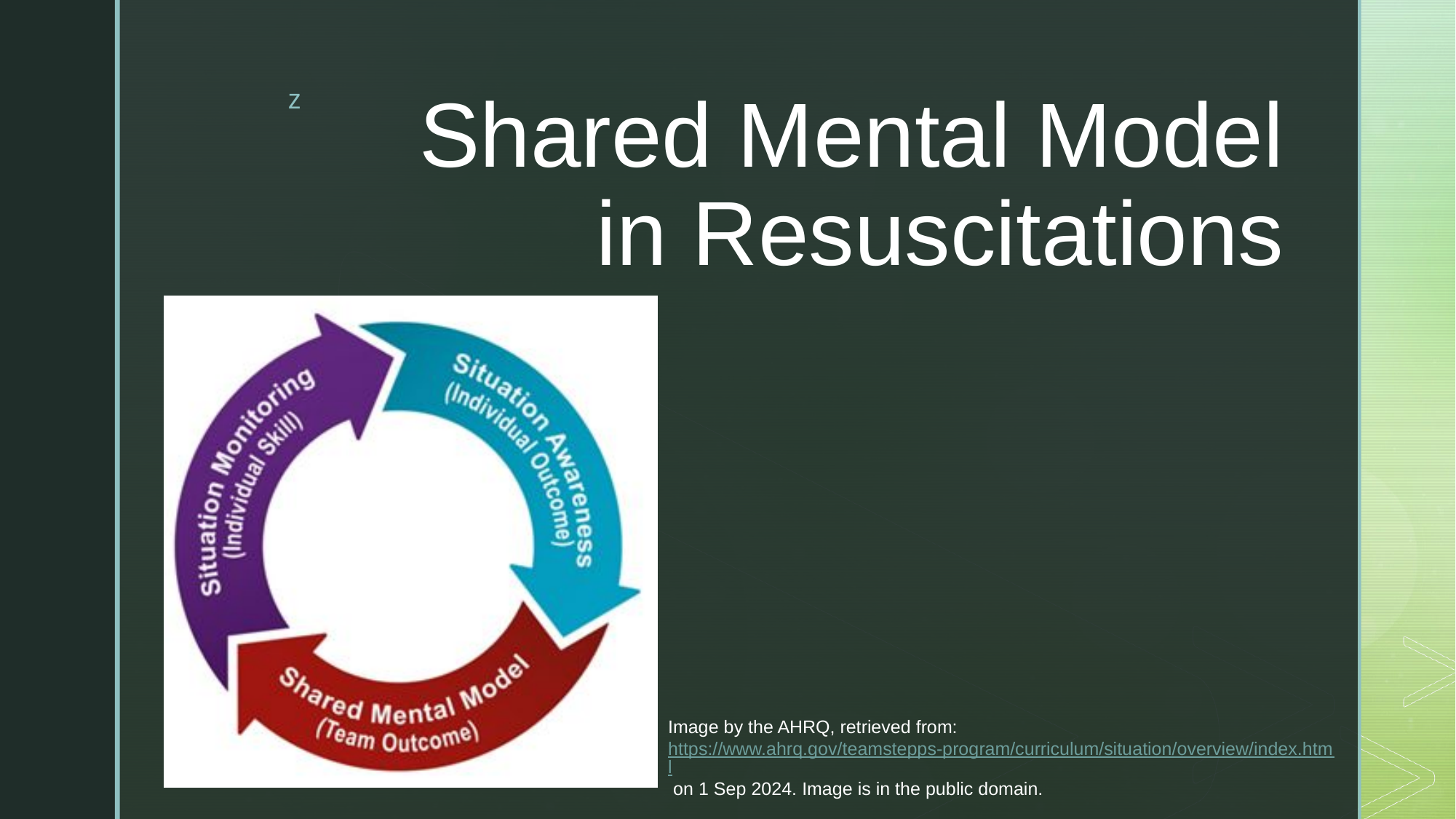

# Shared Mental Model in Resuscitations
Image by the AHRQ, retrieved from: https://www.ahrq.gov/teamstepps-program/curriculum/situation/overview/index.html on 1 Sep 2024. Image is in the public domain.

## Slide 5
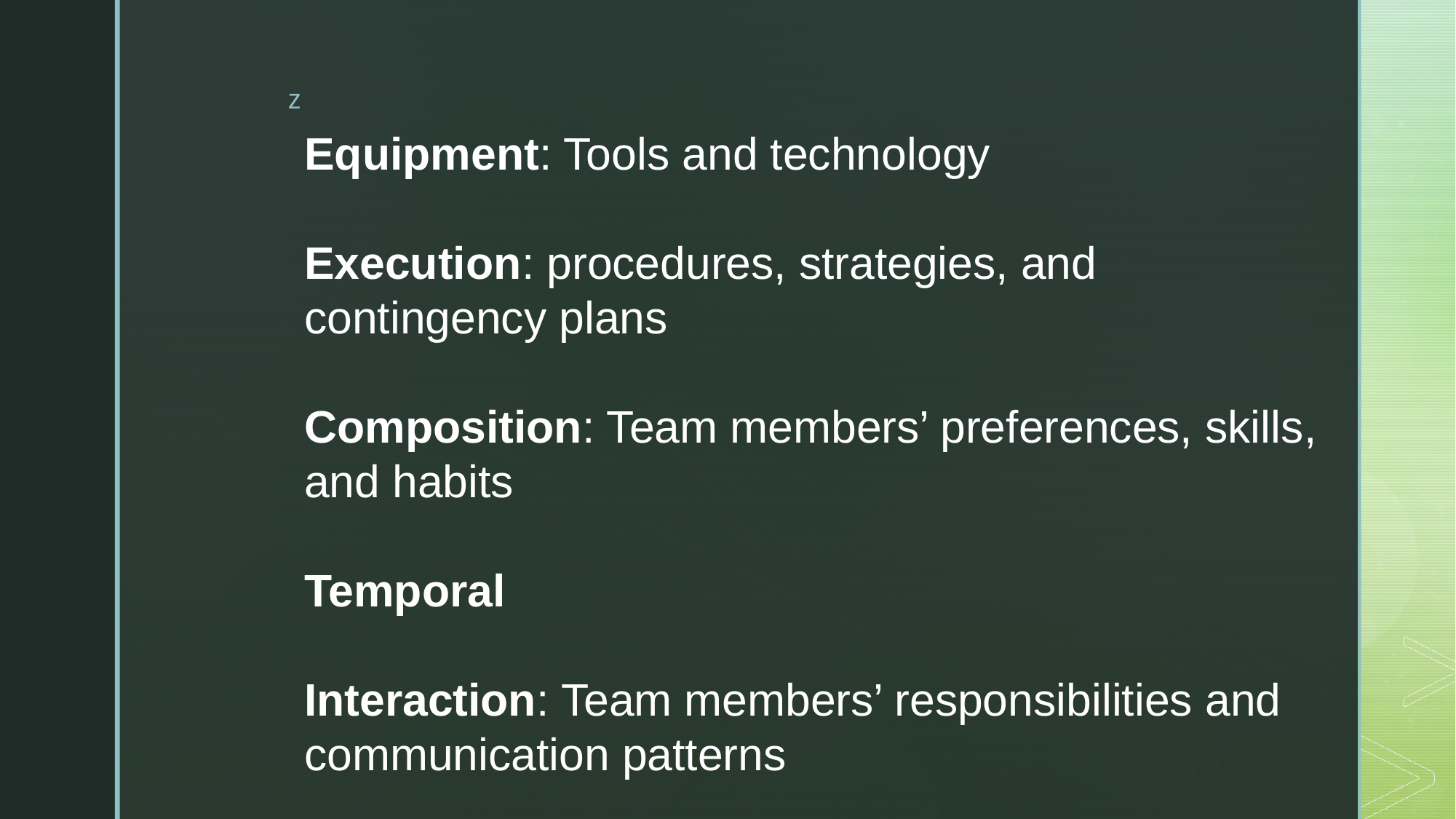

Equipment: Tools and technology
Execution: procedures, strategies, and contingency plans
Composition: Team members’ preferences, skills, and habits
Temporal
Interaction: Team members’ responsibilities and communication patterns

## Slide 6
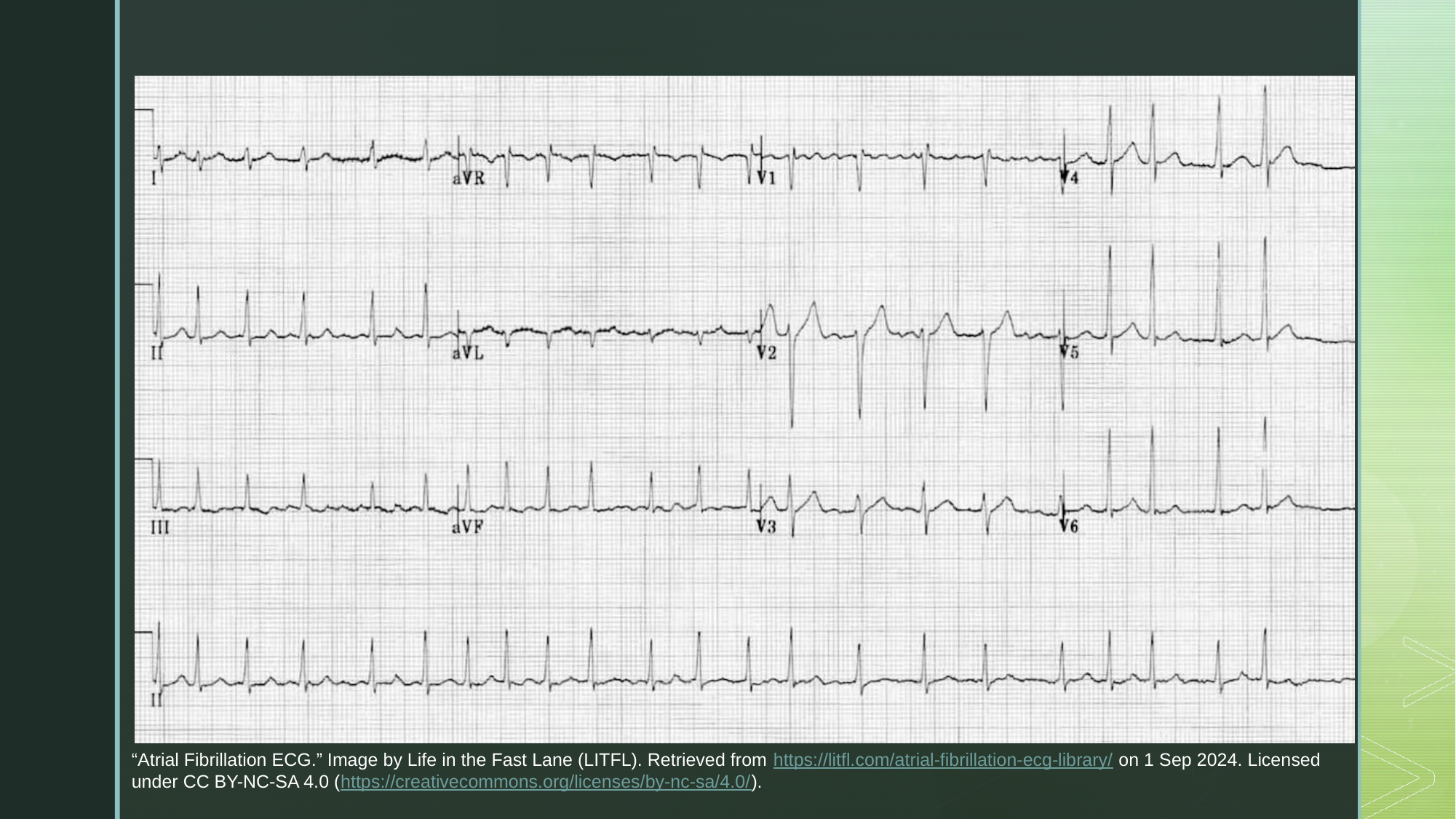

“Atrial Fibrillation ECG.” Image by Life in the Fast Lane (LITFL). Retrieved from https://litfl.com/atrial-fibrillation-ecg-library/ on 1 Sep 2024. Licensed under CC BY-NC-SA 4.0 (https://creativecommons.org/licenses/by-nc-sa/4.0/).

## Slide 7
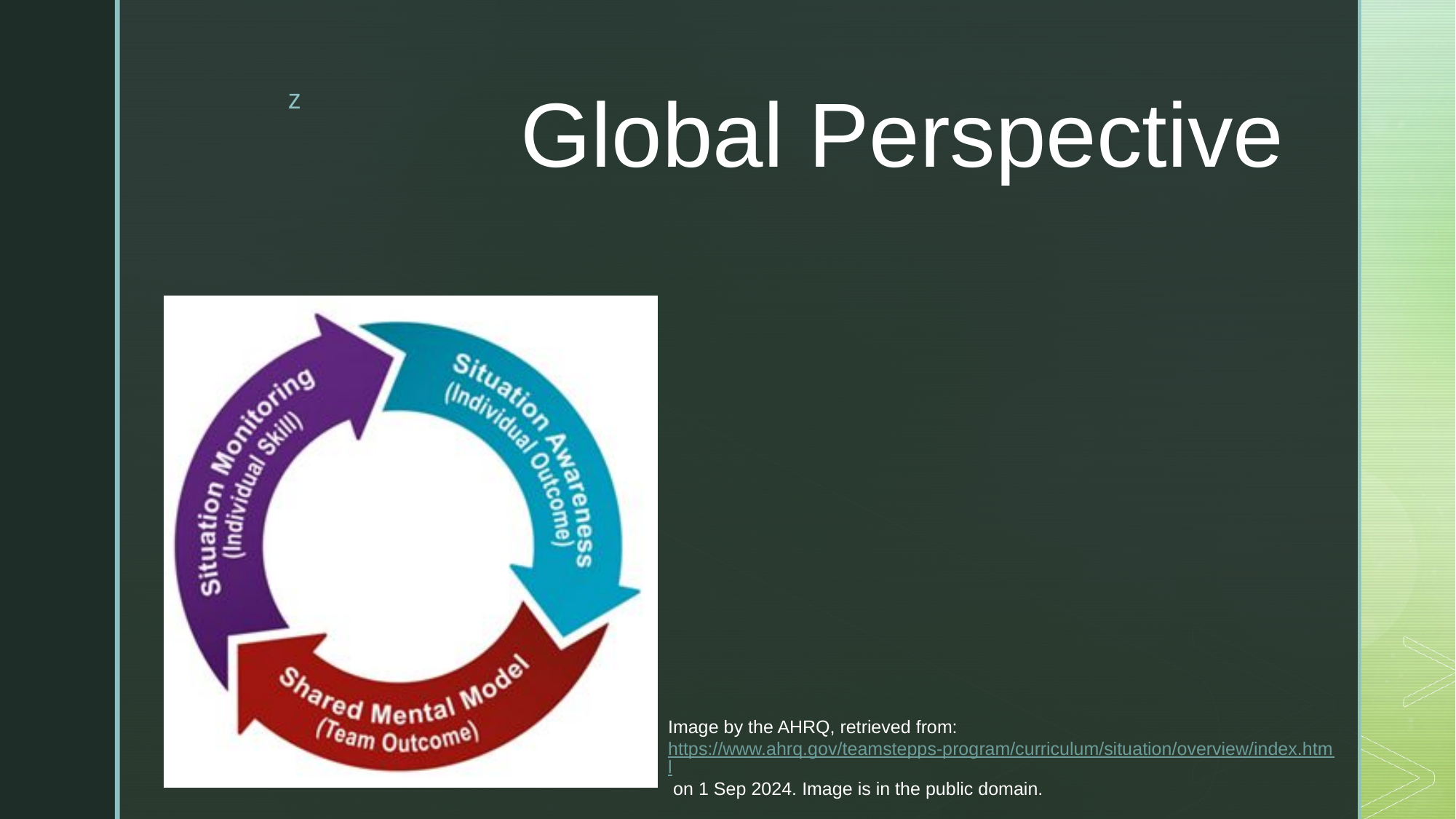

# Global Perspective
Image by the AHRQ, retrieved from: https://www.ahrq.gov/teamstepps-program/curriculum/situation/overview/index.html on 1 Sep 2024. Image is in the public domain.

## Slide 8
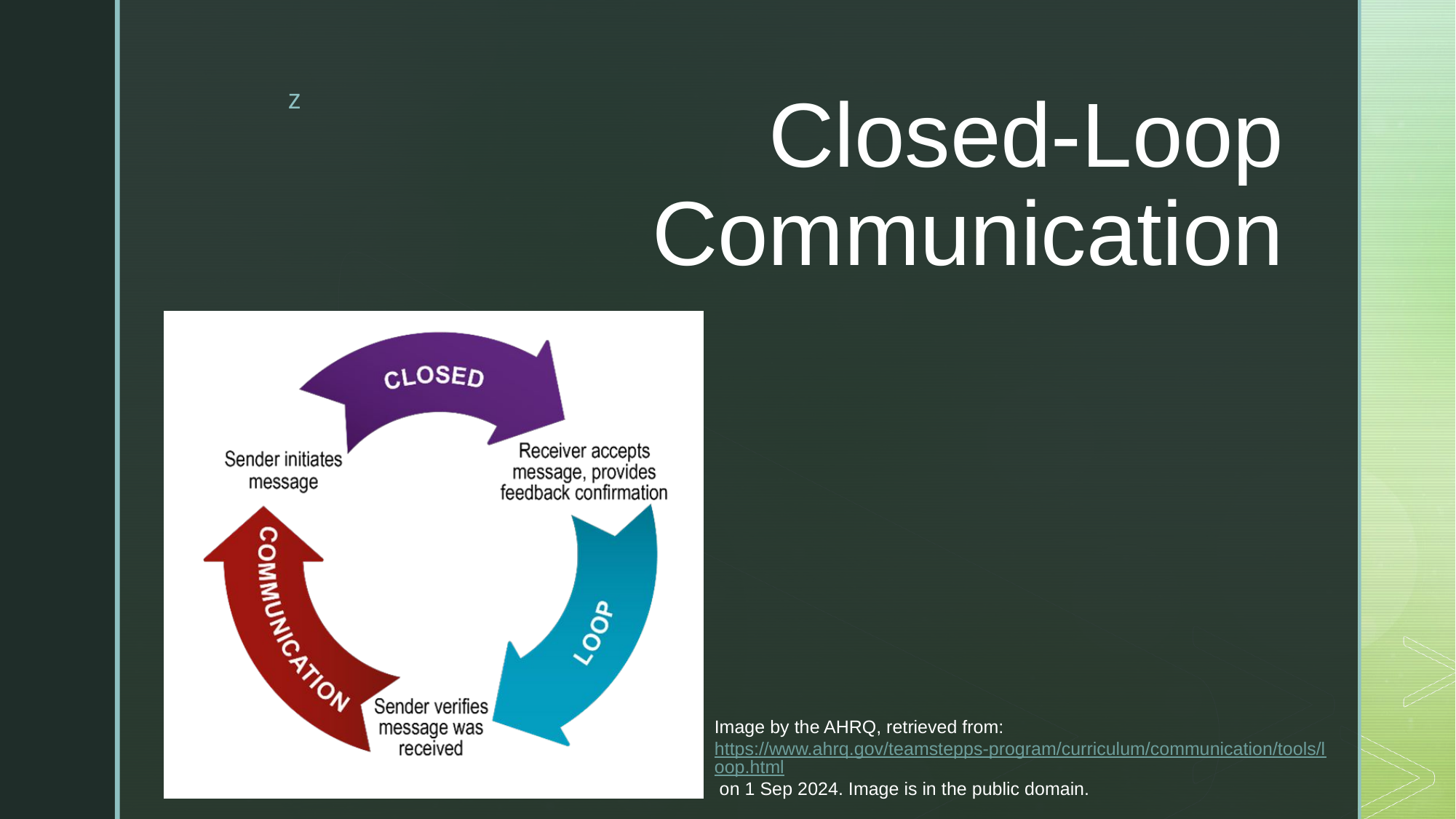

# Closed-Loop Communication
Image by the AHRQ, retrieved from: https://www.ahrq.gov/teamstepps-program/curriculum/communication/tools/loop.html on 1 Sep 2024. Image is in the public domain.

## Slide 9
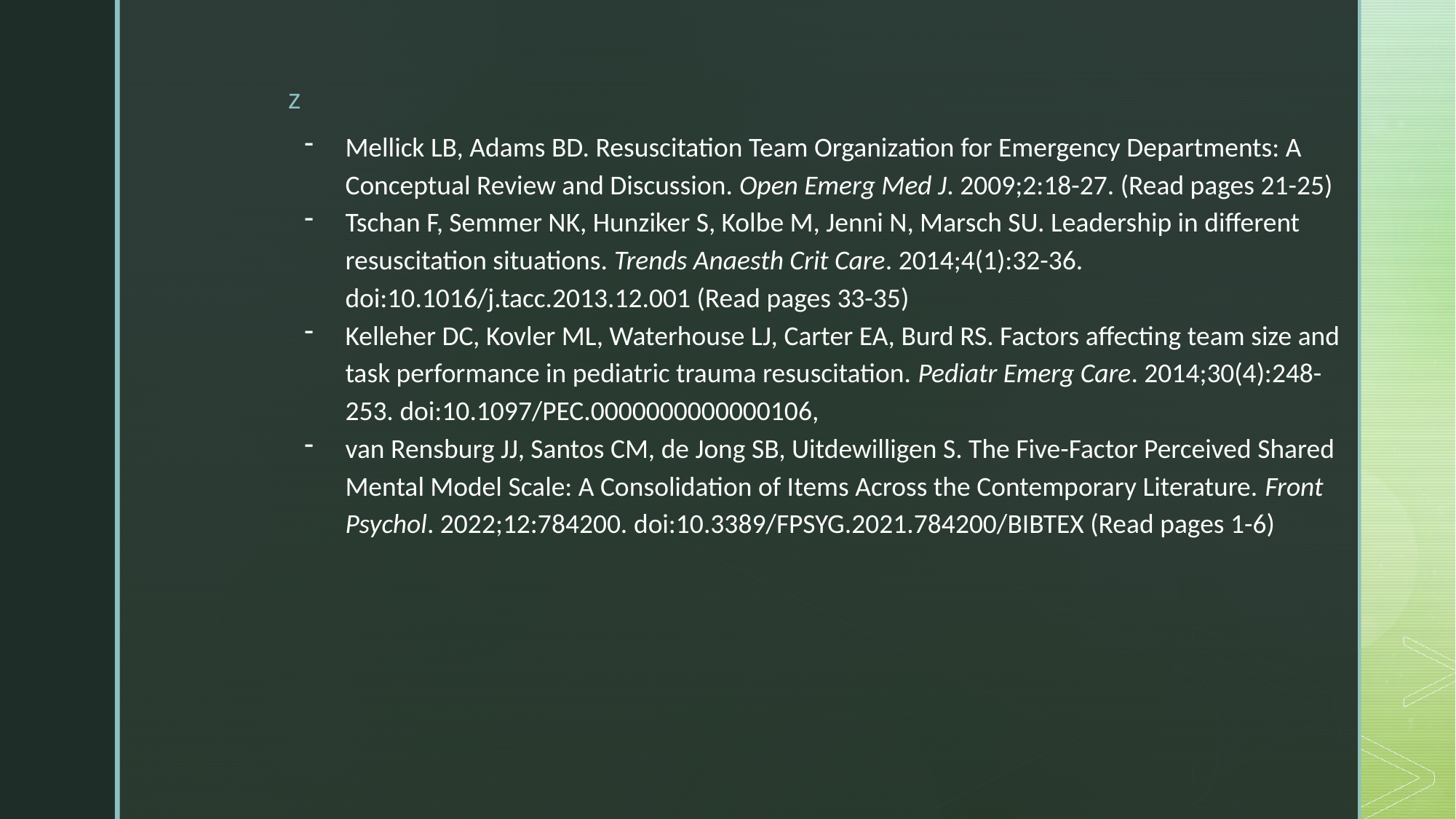

Mellick LB, Adams BD. Resuscitation Team Organization for Emergency Departments: A Conceptual Review and Discussion. Open Emerg Med J. 2009;2:18-27. (Read pages 21-25)
Tschan F, Semmer NK, Hunziker S, Kolbe M, Jenni N, Marsch SU. Leadership in different resuscitation situations. Trends Anaesth Crit Care. 2014;4(1):32-36. doi:10.1016/j.tacc.2013.12.001 (Read pages 33-35)
Kelleher DC, Kovler ML, Waterhouse LJ, Carter EA, Burd RS. Factors affecting team size and task performance in pediatric trauma resuscitation. Pediatr Emerg Care. 2014;30(4):248-253. doi:10.1097/PEC.0000000000000106,
van Rensburg JJ, Santos CM, de Jong SB, Uitdewilligen S. The Five-Factor Perceived Shared Mental Model Scale: A Consolidation of Items Across the Contemporary Literature. Front Psychol. 2022;12:784200. doi:10.3389/FPSYG.2021.784200/BIBTEX (Read pages 1-6)
