# Supplementary material for: An Active-Learning Resuscitation Leadership Curriculum for Emergency Medicine Residents
Source: MedEdPORTAL. 2026 Jun 17;22:11610. doi: 10.15766/mep_2374-8265.11610 (PMC13272583; doi:10.15766/mep_2374-8265.11610)
Supplement: Supplementary file 1 — Resuscitation Leaders Role.docxTeam and Situational Management.docxResuscitation Guidelines and Psychological Safety.docxResuscitation Leaders Role Review.pptxTeam and Situational Management Review.pptxResuscitation Leadership Escape Room.docxFacilitator Overview Guide.docxLBDQ Form.docxPre- and Postsurvey.docx [file mep_2374-8265.11610-s001.zip › F. Resuscitation Leadership Escape Room.docx]

By the end of this session, residents will be able to do the following during a resuscitation:

1. Communicate clear expectations and roles to members of the resuscitation team during emergency department resuscitations.
2. Direct and coordinate team actions during resuscitations by assigning tasks, prioritizing measures, and guiding how interventions should be performed.
3. Apply standardized resuscitation guidelines to guide team decision-making.
4. Maintain clear performance expectations during the resuscitation.
5. Demonstrate leadership behaviors that promote a composed, supportive, and psychologically safe team environment during the resuscitation.

**0:00 - 0:05** Explain the Escape Room and Break into teams

**0:05 - 0:50** Escape Room

**0:50 - 1:00** Team debriefing

**People Needed**

- Lead facilitator (1)
- Small group facilitators (4-6)

**Materials Needed (per group)**

- Discussion Guides
- Handout A: Blank room layout
- Labeled stickers for attending, 1 APP, 2 RN, 1 charge RN, 1 RT, and 1 clerk
- Handout C: environmental scenarios
- Handout D: Closed loop game
- Handout E: Patient monitor
- Handout F: Original ECG with leadership matching game
- Handout G: Repeat ECG with CEASE
- Handout H: DISCERN tool
- Handout I: Letter from family member

Lead Facilitator instructions:

- Prior to the session, ensure all small-group facilitators have the required materials and are familiar with the session objectives and discussion structure. Note that the materials are listed per individual group and enough will need to be prepared based on the number of anticipated groups. Besides printing the handouts, only the labeled stickers will need to be prepared for the session.
- Learners should be broken up into 4-6 groups depending on the number of facilitators available for discussion. Ideal group size is 4–6 learners. Groups may be mixed across PGY levels to promote peer learning and diverse perspectives.
- The lead facilitator will keep time for groups and ensure groups are progressing through the escape room appropriately. Each step of the escape room is anticipated to take 5 minutes maximum.
- At the start of the session, the lead facilitator should explain the following to the entire group, *“Today you will be taking part in an escape room to apply key concepts in resuscitation leadership you have reviewed thus far in this curriculum. You will work together in small groups to progress through a simulated resuscitation scenario by solving a series of challenges. Each step will require you to apply principles of team management, communication, and leadership that have been introduced throughout this curriculum. You are encouraged to think out loud, collaborate with your group, and actively participate in each step. After completing the activity, we will reconvene for a debrief to reflect on both the clinical decisions and leadership behaviors demonstrated during the escape room.”*

**Facilitator Guide**

**Instructions for facilitators**: Today you will be running a resuscitation escape room activity. You will guide learners through a simulated resuscitation scenario in which they must complete a series of tasks (“puzzles”) before progressing to the next stage. **A medical education escape room is an interactive, team-based learning activity in which learners apply clinical knowledge, decision-making, and communication skills to solve sequential challenges that advance a case scenario. In this activity, learners will apply resuscitation leadership concepts introduced throughout this curriculum to progress through a simulated resuscitation. Each stage of the escape room should take approximately 5 minutes, for a total of 45 minutes of gameplay, followed by a 10-minute facilitated debrief.**

The discussion guide is set up as follows:

- **Patient status**- Provides a brief description of the clinical situation and where learners are in the resuscitation. This column is for facilitator reference only and should not be read aloud to learners.
- **Escape room sequence-** This is the scenario script. Read this aloud to learners to introduce each stage and frame the task.
- **If Learners do this…-** Describes the expected actions or solutions for each stage and prompts for struggling learners. This serves as the facilitator answer guide and should not be shared directly with learners.
- **Then this will happen**- Provides the outcome of learner actions, including what information, prompts, or handouts to give next. Use this to advance the scenario.

The goal of this activity is to review and apply the core objectives of the resuscitation leadership curriculum. **Learners should be encouraged to verbalize their reasoning and work as a team when solving each task.** If learners are having difficulty, you may provide **brief, targeted hints to maintain progression**, but allow learners adequate time to attempt each step independently before intervening. **At the conclusion of the escape room, facilitators should transition directly into the structured debrief using the provided debriefing questions. This debrief is a critical component of the learning experience.**

**If a group becomes significantly stuck (>2–3 minutes without progress), provide a prompt to help redirect them.** **Facilitators should also ensure balanced participation, encouraging quieter or more junior learners to contribute to decision-making and task completion.** Should you have questions during the activity, please wave over the lead facilitator for assistance.

Table of Contents:

Materials Outline- 3

Answer Guide- 4

Escape Room Guide- 5-7

Debriefing Questions- 7

**Materials by step:**

1. A blank room layout that says “IM SAFE, beat the stress fool”
2. Labeled stickers for the attending physician, an APP, 2 nurses, 1 charge nurse, 1 respiratory therapist, and 1 clerk (7 stickers per group)
3. Sheet with following scenarios: A) The video laryngoscope is broken. B)You do not have any central access supplies in the room. C)Your adaptor for your defibrillator to the pads is going to be different from EMS’s D) the MICU team is standing at the edge of the room loudly talking about their plan for the incoming patient. (4 flashcards per team)
4. Closed loop game: Paper with three circular prompts of closed-loop communication
5. Patient monitor: The patient monitor shows CPR rhythm with a rate of 80, no blood pressure, SPO2 is 99% with BVM, and ETCO2 is 15.
6. Original ECG with leadership matching game
7. Repeat ECG with Vfib arrest, on the back is CEASE with space below for learners to write in what the mnemonic stands for.
8. Debriefing tool
9. Letter from family member

**Answer Guide:**

1. Zero-Point Survey- STEP-UP (self, team, environment, primary survey, update, priorities.
   1. IM SAFE- When assessing teams' ability to perform, consider illness, medications, stress, alcohol(drugs), fatigue, and eating/elimination.
   2. Beat The Stress Fool- Breathe, Talk, See (mental rehearsal), Focus (trigger word).
2. All 7 stickers should be placed in the room (clerk can be placed outside of the room)
3. Example response: For laryngoscope, they can set up direct and/or cric. For no central access supplies they can find an IO gun in the room, have nurses prepare large bore IVs, or ask a team member to leave the room and find equipment. Your team can ask EMS to leave the defibrillator on or swap pads as soon as the patient arrives. Kindly and respectfully ask the MICU team to step outside and quiet down.
4. Example:
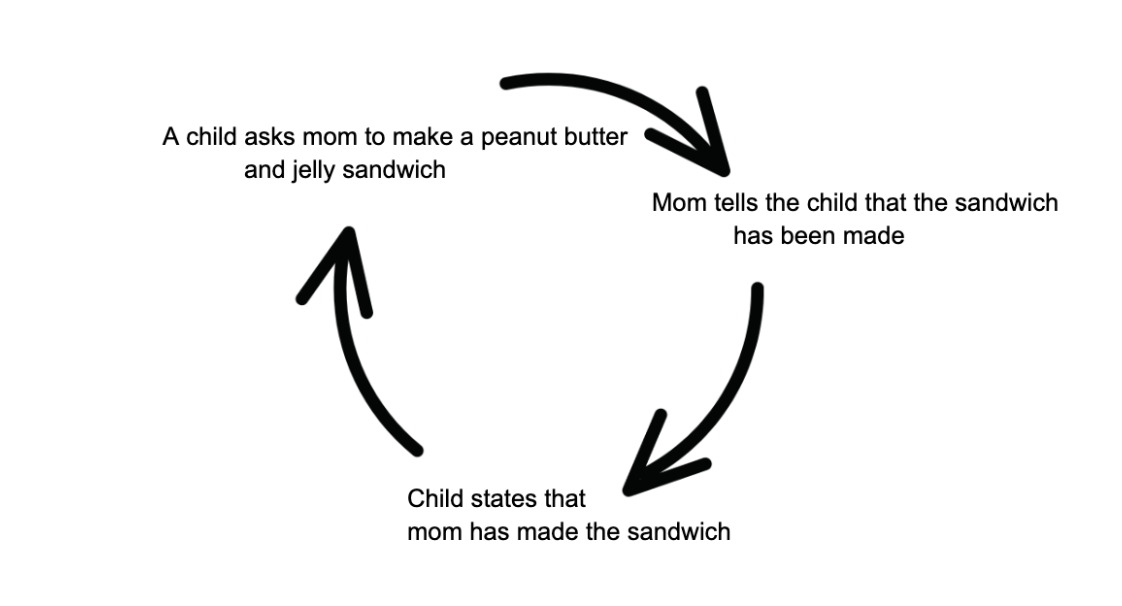

5. The learner, recognizing poor CPR quality (low HR) needs to either ask about CPR quality, notice the low HR and coach improved quality CPR, or ask for improved quality CPR/rotating of compressor
6. Per scenario
7. Your senior charge nurse- **empowering** leadership (highly experienced nurse conducting high-level activity does best with empowering leadership, allows the nurse to use their experience unabated).
8. A nurse who you have worked closely with for the past 5 years- **empowering** leadership (highly experienced nurse conducting mid to low-level activity does best with empowering leadership, allows the nurse to use their experience)
9. A new hire nurse directly out of nursing school- **directive** leadership (new hire nurse conducting high-risk activity requires more hands-on and directive leadership to ensure the high-risk task is being done correctly)
10. Respiratory Therapist- **empowering** (new hire nurse conducting low-risk activity- given the low-risk nature there is little risk of using empowering leadership while directive leadership may distract team leader from more important interventions).
11. **Clinical Features**, **Effectiveness** of ongoing interventions, **Ask** if anyone else has any ideas, **Stop** the Resuscitation, and **Explain** to family.
12. Have the team run through the debriefing questions thoughtfully.
13. Example def- The act of guiding a team during life-threatening medical emergencies to optimize patient outcomes

**Escape Room Guide**

| **Patient Status (What learners should know at this stage)** | **Escape Room Sequence (Facilitator Actions & Script)** | **If Learners Do This… (Expected Actions)** | **Then This Will Happen (Facilitator Response)** |
| --- | --- | --- | --- |
| 1. Pre-arrival: Patient en route in cardiac arrest (10 minutes away). Learners are given a blank room layout labeled “STEP-UP.” | EMS radio call: “This is Ferndale Fire coming in with priority I traffic for a cardiac arrest. We have a 56-year-old male with witnessed cardiac arrest at his home. The family saw the patient collapse in the kitchen while he was cooking. They started CPR immediately. We took over and continued CPR following ACLS protocol. We will be at your facility in 10 minutes.”  Ask the team, “How do you want to get ready?” | The team should follow the zero-point survey by completing the Self component. This can be done either by reciting the “IM SAFE” mnemonic (self, team, environment, primary survey, update, priorities), “Beat the Stress, Fool” mnemonic (Breathe, Talk, See (mental rehearsal), Focus (trigger word), or by discussing how they will physically and cognitively prepare themselves for this resuscitation.  If the learners struggle, prompt: “How are you preparing yourself before the patient arrives?” | If learners demonstrate preparation (any structured approach), provide team member stickers. |
| 1. Pre-Arrival: The patient is still inbound, with no updates. The team is assembled | “Your team has now arrived. You are serving as the attending physician. With you are an APP, 2 nurses, 1 charge nurse, 1 respiratory therapist, and 1 clerk. Please prepare them for this resuscitation” | Learners should assign roles and physically place team members on the room layout while defining their responsibilities.  If the learners struggle, prompt “Who is responsible for airway? Medications? Documentation?” | If roles are clearly assigned, given environmental hazard page (handout C). (Note- this can be given as one page or as individual cards). |
| 1. Pre-Arrival: Environmental challenges identified. | “As you prepare for the patient and look around the room you realize there are issues with some of the equipment. What will you do?” | The team should discuss and write how they will deal with each environmental danger. Acceptable solutions include backup airway preparation, IO gun prepared, use EMS defibrillator equipment, and and respectfully ask the MICU team to step away.  If learners struggle, prompt “What is your backup plan?” | If environmental concerns are addressed, the patient arrives, give the closed-loop game (handout D). |
| 1. Arrival: Patient arrives in cardiac arrest with ongoing CPR. | EMS arrives and says: “Hi team. So this is a 56-year-old male who collapsed today. We have been doing CPR for 15 minutes after the family did it for about 10. He is bagging easily with BVM. The family will be here soon with more information.”  “It’s time to continue resuscitating this patient. Using closed-loop communication, instruct your team on how to proceed.” | Learners must demonstrate 3 closed-loop communication exchanges (order-> acknowledgement-> confirmation).  If learners struggle, prompt “Can you confirm the task was completed?” | If exchanges are detailed (ex: give epinephrine, epinephrine given, confirmed epinephrine given), give the team a picture of the patient’s monitor (Handout E) |
| 1. Active resuscitation: The patient is hooked up to the monitor. | “The patient monitor is as shown during CPR. Your nurse is concerned about the vitals. How will you intervene?” | Learners should recognize inadequate CPR (HR of only 80) and correct compression quality.  If the learners struggle:   - After 2 minutes state that a nurse is uncomfortable with the chest compressor’s technique. - After 4 minutes, state that not doing CPR at 100-120 compressions per minute is a safety concern. | If CPR quality concern raised or compressor changed, give the team an EKG and matching game (Handout F) |
| 1. Active Resuscitation: Vfib identified. | “The patient’s rhythm is show on your handout. Respond to the leadership scenarios on the back of the rhythm strip to defibrillate the patient” | Learners complete the leadership match on the back of handout F  If the learners struggle, prompt “What is the definition of empowering leadership? What is the definition of directive leadership?” | When the learners correctly answer each scenario (new hire nurse directive leadership, all others empowering) state “the patient has been defibrillated” and hand them handout G. |
| 1. Active resuscitation: Persistent Vfib after 30 minutes, family arrives. | “The patient remains in pulseless Vfib despite repeated defibrillations and appropriate care for the last 30 minutes. The family of the patient, identified as Ceaser Doe, are now present and quite distressed. What do you do next?” | Learners should apply the CEASE framework to consider stopping the resuscitation.  If the learners struggle, the facilitator should clue the learners to each step of the CEASE mnemonic (patient's **Clinical Features**, **Effectiveness** of interventions, **Ask** for ideas, **Stop** resuscitation, **Explain** to family). | If learners correctly address each component of CEASE, the patient will be defibrillated to NS and the case will move to a debriefing (handout H). |
| 1. ROSC: Patient up to ICU | “A nurse suggests a vector change and the patient achieves ROSC. They are transferred to the MICU in critical condition. You gather your team to debrief the resuscitation.” | Learners complete the structured debriefing. | When learners thoughtfully address each part of the debriefing, provide them with handout I. |
| 1. Post-event reflection: Family note | “A family member has written a note for your team.” | Learners define resuscitation leadership. | Proceed to debriefing questions (if time allows) |

Debriefing questions:

- What went well during this game?
- What could have been improved?
- What do you view your role as during resuscitations in the next 6 months? Next year? In five years?
- How do you plan to use the knowledge and skills you’ve learned from this curriculum in your practice?

Handout A

**IM SAFE, Beat the Stress Fool**


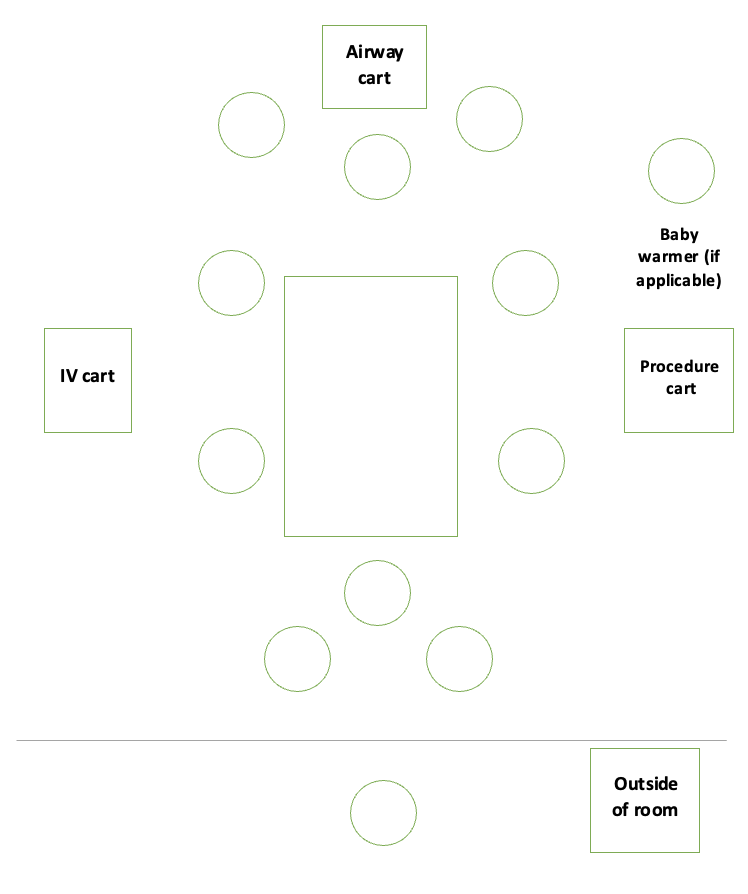


Author Owned

Handout C

| The video Laryngoscope is broken  ____________________________________ | There are no central access supplies in the room  ____________________________________ |
| --- | --- |
| The adaptor for the defibrillator to the pads is different from EMS  ____________________________________ | The MICU team is standing at the edge of the room yelling loudly about another patient  ____________________________________ |

Handout D


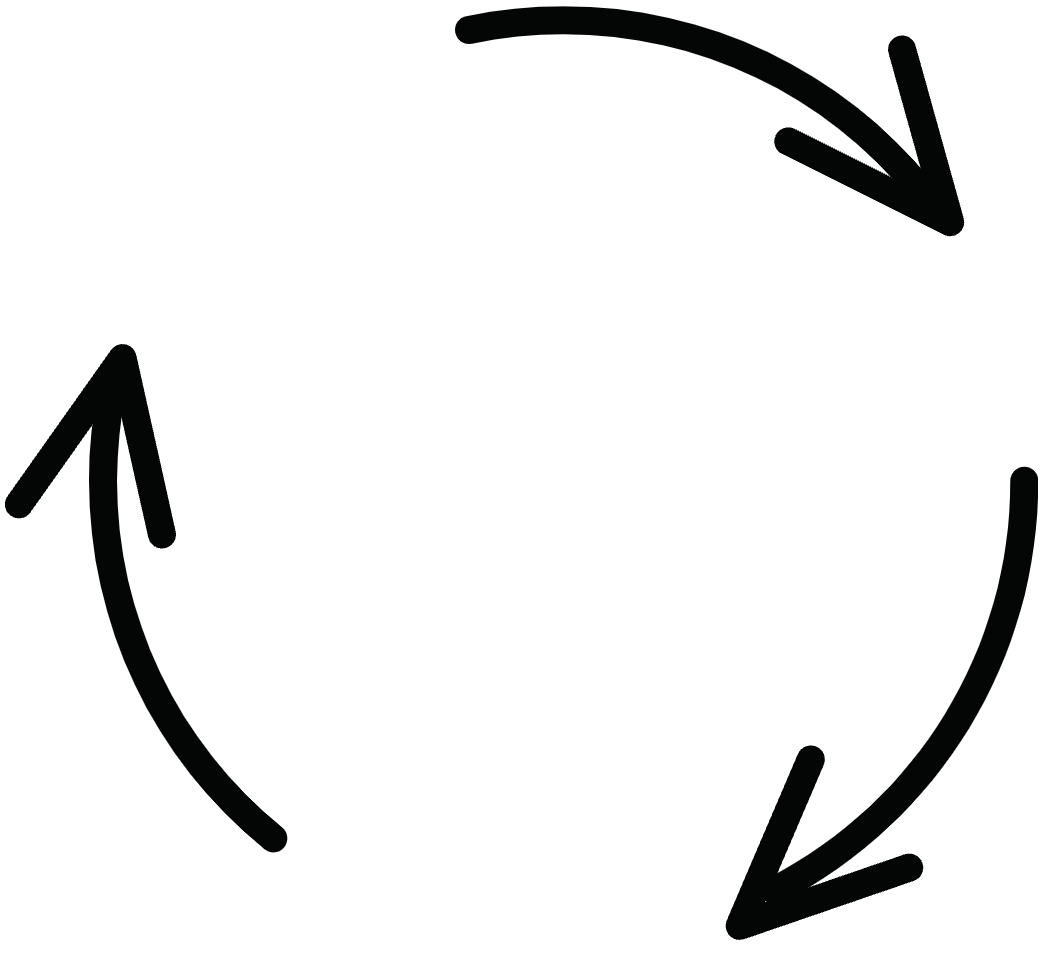


—----------------------------------------------------------------------------------------------------------------------------


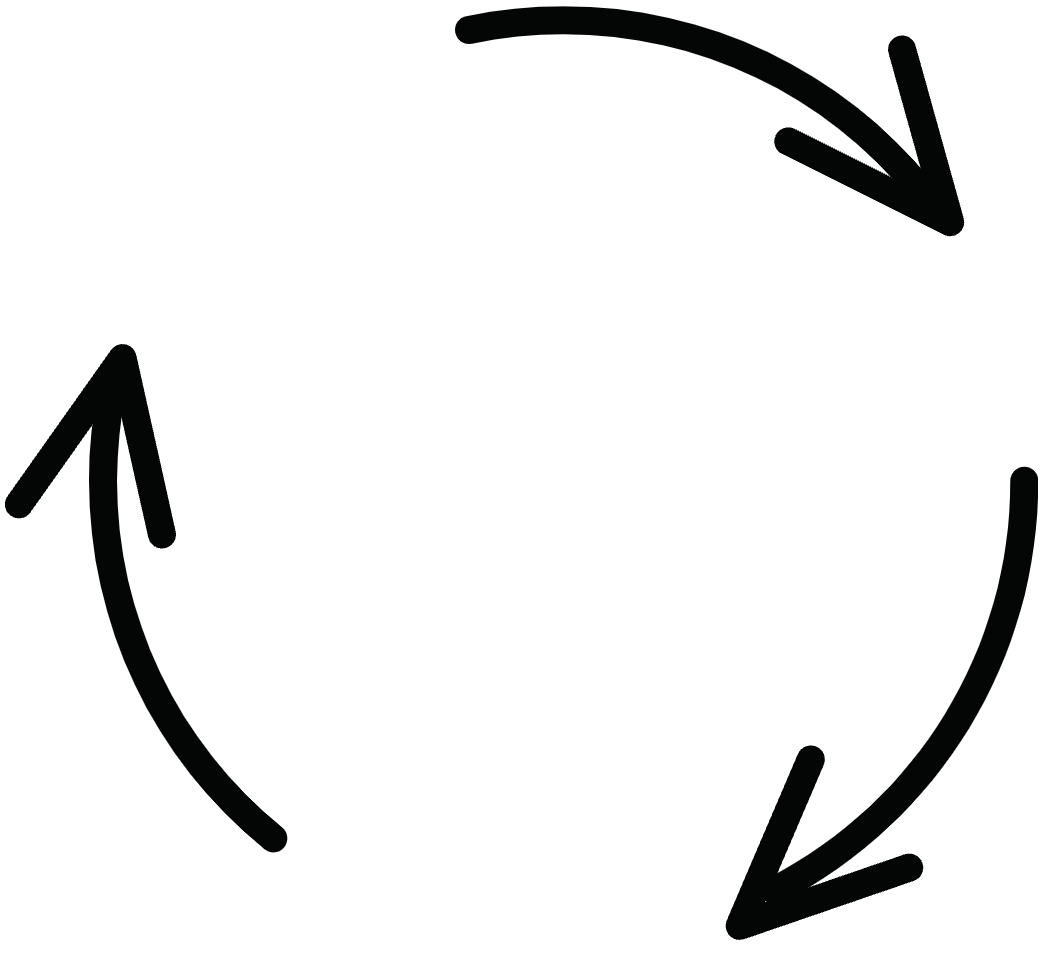


—----------------------------------------------------------------------------------------------------------------------------


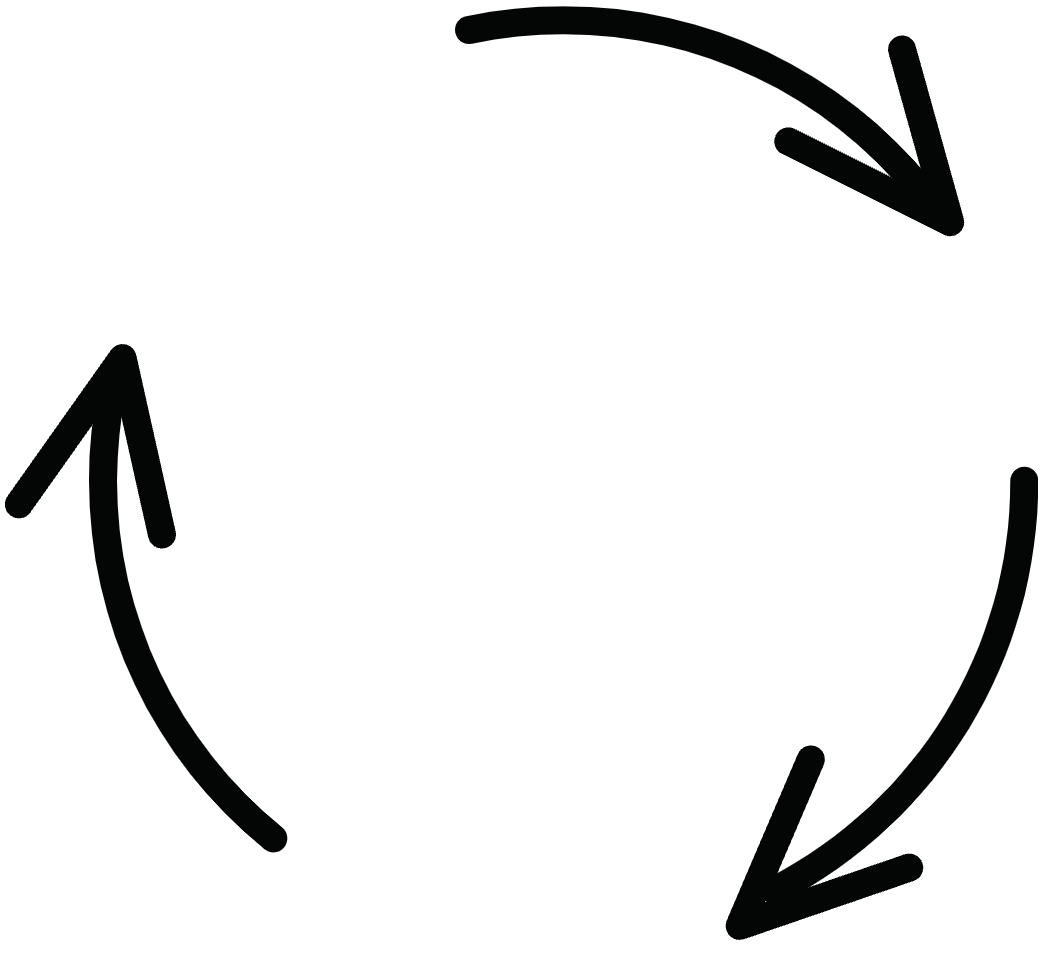


Handout E


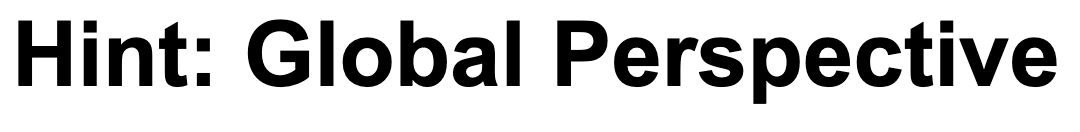


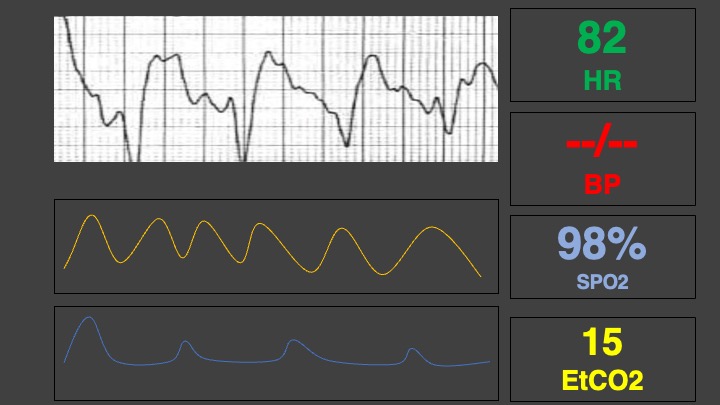


Rhythm strip of image adapted from “ECG Motion Artefacts.” Life in the Fast Lane (LITFL), <https://litfl.com/ecg-motion-artefacts-ecg-library/>. Licensed under CC BY-NC-SA 4.0 (<https://creativecommons.org/licenses/by-nc-sa/4.0/>). Remaining figure elements created by the author.

Handout F(1)

**Directive or Empowering?**

**(see back)**


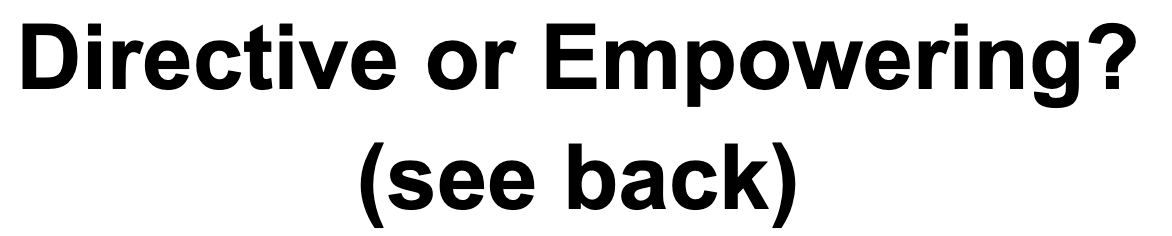


**
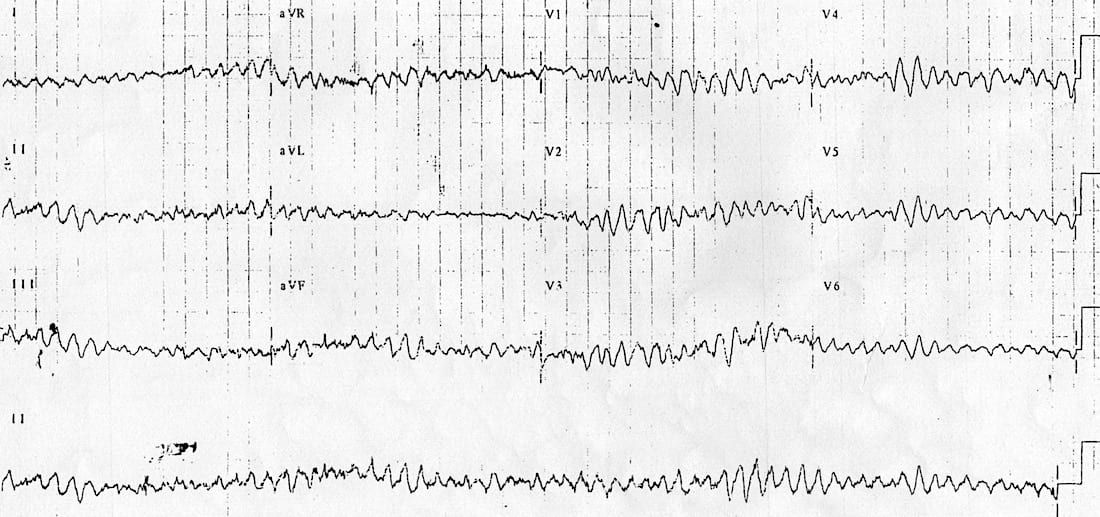
**

“Ventricular Fibrillation (VF) ECG.” Image by Life in the Fast Lane (LITFL). Retrieved from <https://litfl.com/ventricular-fibrillation-vf-ecg-library/> on 1 Nov 2024. Licensed under CC BY-NC-SA 4.0 (<https://creativecommons.org/licenses/by-nc-sa/4.0/>).

Handout F(2)

**Directive or Empowering?**

| 1. Your senior charge nurse who you charge with getting additional IV access and giving medications.   ____________ | 1. A nurse who you have worked closely with for the past 5 years and who you charge with documenting time course and interventions during the code.   ____________ |
| --- | --- |
| 1. A new hire nurse directly out of nursing school who you charge with placing defibrillator pads for dual sequential defibrillation and running the defibrillator.   ____________ | 1. A relatively new hire respiratory therapist new to your institution who you charge with bagging the patient.   ____________ |

Handout G

**Repeat ECG: CEASEr Doe**

**C___ E___ A___ S___ E___**
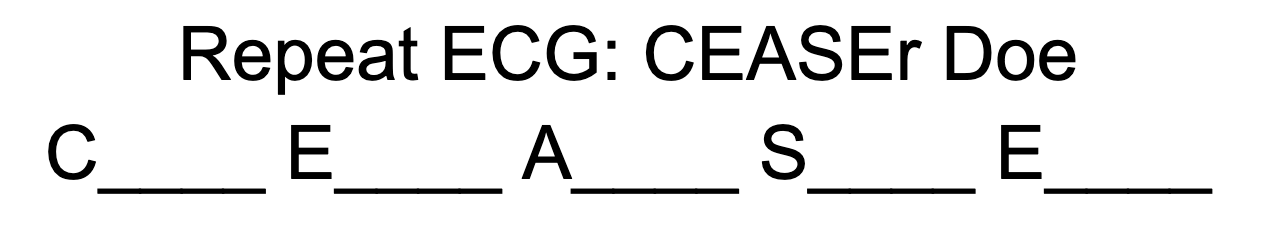


**
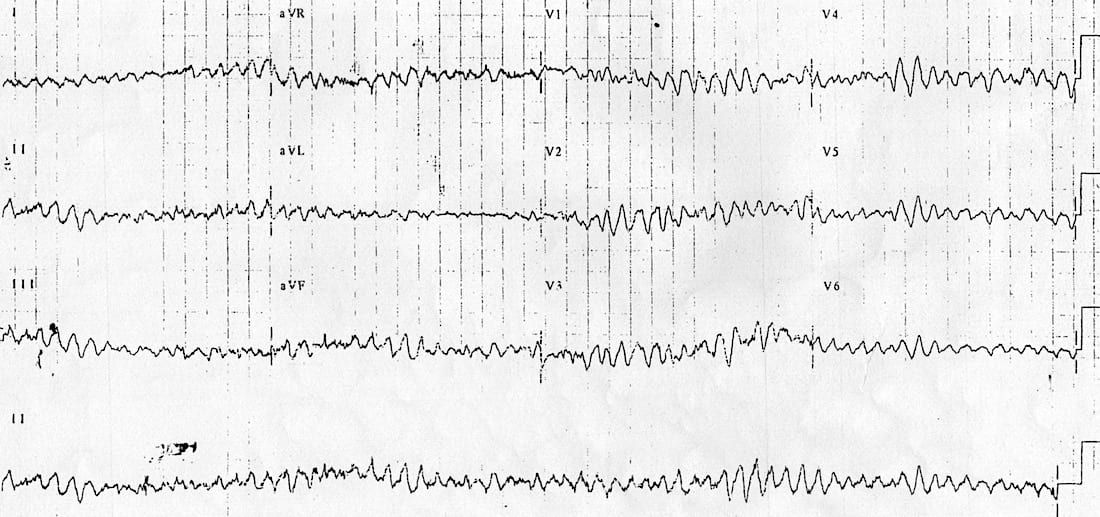
**

“Ventricular Fibrillation (VF) ECG.” Image by Life in the Fast Lane (LITFL). Retrieved from <https://litfl.com/ventricular-fibrillation-vf-ecg-library/> on 1 Nov 2024. Licensed under CC BY-NC-SA 4.0 (<https://creativecommons.org/licenses/by-nc-sa/4.0/>).

Handout H

The purpose of debriefing is for education, quality improvement, and emotional procesing: it is not intended to be a blaming session. These debriefings usually take several minutes. Let’s review these questions so that we as an entire team can discuss what went well and what could have gone better.

| **What went well during our care for the patient?** |
| --- |
| **What could have improved during our care for the patient?** |
| **What are potential solutions?** |
| **Was the team leader the only provider calling out orders?** |
| **Was anyone confused about who was the team leader?** |

Handout I

To the ER Doctors,

You took care of my family member Ceaser Doe recently who had come in after his heart stopped. I cannot thank you all enough for your hard work and dedication. My Ceaser is alive thanks to you. I was especially impressed at how you managed your team and worked so well together. I was wondering if you could tell me what it was exactly you were all doing?

What is Resuscitation Leadership?

___________________________________

___________________________________

___________________________________
