# Supplementary material for: An Active-Learning Resuscitation Leadership Curriculum for Emergency Medicine Residents
Source: MedEdPORTAL. 2026 Jun 17;22:11610. doi: 10.15766/mep_2374-8265.11610 (PMC13272583; doi:10.15766/mep_2374-8265.11610)
Supplement: Supplementary file 1 — Resuscitation Leaders Role.docxTeam and Situational Management.docxResuscitation Guidelines and Psychological Safety.docxResuscitation Leaders Role Review.pptxTeam and Situational Management Review.pptxResuscitation Leadership Escape Room.docxFacilitator Overview Guide.docxLBDQ Form.docxPre- and Postsurvey.docx [file mep_2374-8265.11610-s001.zip › G. Facilitator Overview Guide.docx]

**Overview**

This appendix serves as a consolidated implementation guide to support **lead and small-group facilitators in delivering** this resuscitation leadership curriculum. It was created based on the experience and feedback of the initial implementation of the curriculum. It is designed to enhance curriculum adoption and **support facilitators in session delivery**, including best practices for active learning, gamified strategies, and small-group discussion management. **This guide is organized into two sections: (1) lead facilitator instructions and (2) small-group facilitator resources.**

**Lead Facilitator Information:**

The curriculum is designed as a longitudinal series with sessions building on previous topics. Sessions are intended to be delivered in sequence. However, individual components may be adapted based on local needs.

Curriculum instruction:

- The curriculum is delivered using the flipped classroom model, with learners reviewing pre-reading prior to sessions and engaging in active learning discussions for each session.
- Pre-reading material should be disseminated to learners at least 2 weeks prior to each session. Pre-reading is anticipated to take 30 minutes.
- Pre-reading completion is encouraged but not formally assessed; therefore, sessions include brief review components to ensure all learners can participate meaningfully regardless of preparation.
- Sessions are designed for small groups (4–6 learners per small-group facilitator) with mixed postgraduate years to promote peer teaching, expose junior learners to advanced reasoning, and allow senior learners to practice leadership and teaching skills.
- Session facilitators may be faculty, fellows, or senior residents. While we recommend facilitators be oriented to curriculum materials, senior resident facilitators should have additional leadership training and receive a brief orientation prior to sessions to review objectives, flow, and facilitation strategies.
- Sessions should be hosted in rooms where groups can physically separate to minimize distractions.
- For each session, included materials are specified per appendix. Learners will need printed handouts and writing utensils. Facilitators will require a discussion guide and any session-specific materials.

**Small Group Facilitator Information:**

Role: Your role as a small group facilitator is to guide learners through the session, as opposed to lecturing. Your main priority during discussion is to highlight leadership behaviors during resuscitations as opposed to clinical-decision making. Your responsibilities will include

- Maintaining time and session flow
- Encouraging participation from all learners
- Redirect discussion toward leadership and communication concepts
- Provide prompts or hints when learners struggles.

Facilitators should prioritize discussion of team dynamics, communication, and leadership behaviors rather than focusing exclusively on clinical management. Below, we will go into additional detail of these responsibilities

Role assignment: During role playing and case-based scenarios, ensure roles are assigned to volunteers at the start of each case. Prioritize junior learner involvement when possible. **If junior learners do not volunteer, facilitators should directly invite participation or use a rotation system across cases to ensure equitable leadership opportunities.**

Managing Group Dynamics: During discussion, monitor group interactions. Encourage quieter learners to contribute, redirect dominant learners, and promote respectful discussion and psychological safety. For challenging learners, use open-ended questions, refocus discussion on leadership principles, and invite alternative perspectives from the group. The lead facilitator will always be available should redirection fail.

Active Learning Strategies: There are four primary methods of active learning utilized in this curriculum

- Flipped classroom- learners review key content prior to sessions, allowing in-session time to focus on application
- Case-based learning- realistic scenarios promote contextual application of knowledge
- Role-play: Allow learns to practice communication and leadership behaviors
- Gamification (escape room): Use game rules and methods to enhance engagement and reinforce learning through interactive challenges.

Preparation: Prior to each session, you should review the entirety of the discussion guide and handouts. Learners are provided with pre-reading material for each session which is summarized in these documents. While not required, you are encouraged to review the pre-reading material prior to your discussions.

In-session tips:

- Keep track of time as prompted by the lead facilitator. Aim to keep group on pace while allowing flexibility for meaningful discussion. If your group falls behind, provide targeted prompts and advance the cases to maintain overall session flow.
- During discussion, encourage reflection of actions and thought processes. Highlight and reinforce learning objectives.
- If discussion becomes overly focused on diagnosis or clinical management, redirect learners to reflect on leadership, communication, and team coordination.

Facilitators interested in additional background on active learning, flipped classrooms, and gamification in medical education may find the following resources helpful:

- **Active Learning & Small Group Facilitation:**
  Wolff M, Wagner MJ, Poznanski S, Schiller J, Santen S. *Not another boring lecture: Engaging learners with active learning techniques.* J Emerg Med. 2015.¹
- **Flipped Classroom:**
  Lew EK. *Creating a contemporary clerkship curriculum: the flipped classroom model in emergency medicine.* Int J Emerg Med. 2016.²
- **Gamification & Escape Rooms:**
  Gue S, Ray J, Ganti L. *Gamification of graduate medical education in an EM residency program.* Int J Emerg Med. 2022.⁴
  Shah AS, Pitt M, Norton L. *Escape the Boring Lecture: Tips and Tricks on Building Puzzles for Medical Education Escape Rooms.*
- **General Active Learning Strategies:**
  Fornari A, Poznanski A (eds). *IAMSE Manual: Active Learning: How-to Guides for Health Profession Educators.*
