# Supplementary material for: An Active-Learning Resuscitation Leadership Curriculum for Emergency Medicine Residents
Source: MedEdPORTAL. 2026 Jun 17;22:11610. doi: 10.15766/mep_2374-8265.11610 (PMC13272583; doi:10.15766/mep_2374-8265.11610)
Supplement: Supplementary file 1 — Resuscitation Leaders Role.docxTeam and Situational Management.docxResuscitation Guidelines and Psychological Safety.docxResuscitation Leaders Role Review.pptxTeam and Situational Management Review.pptxResuscitation Leadership Escape Room.docxFacilitator Overview Guide.docxLBDQ Form.docxPre- and Postsurvey.docx [file mep_2374-8265.11610-s001.zip › H. LBDQ Form.docx]

Resident:_________________________ Evaluator: _________________________

**Leadership Behavior Description Questionnaire**

Instructions:

1. READ each item carefully
2. THINK about how frequently the leader engages in the behavior described by the item
3. DECIDE whether he/she/they acts as described by the item **(A) Performed, (B) Partially Performed, (C) Not Performed**
4. PUT AN X IN THE COLUMN of the corresponding assessment following the item to show the answer you selected

|  | **Performed** | **Partially Performed** | **Not Performed** |
| --- | --- | --- | --- |
| 1. The leader let the team know what was expected of them (through direction and command) |  |  |  |
| 2. The leader demonstrated the use of uniform guidelines |  |  |  |
| 3. The leader displayed a positive attitude |  |  |  |
| 4. The leader decided what should be done |  |  |  |
| 5. The leader decided how things should be done |  |  |  |
| 6. The leader assigned group members to particular tasks |  |  |  |
| 7. The leader made sure that their part in the team was understood by the team members |  |  |  |
| 8. The team leader planned the work to be done |  |  |  |
| 9. The team leader maintained definite standards of performance |  |  |  |

Optional Comments: **____________________________________________________________**

**______________________________________________________________________________**

**If form not completed, please state why: ___________________________________________**

*(Please hand to resus resident or place in the resident evaluation lock box in the C workroom once completed)*
