# Supplementary material for: An Active-Learning Resuscitation Leadership Curriculum for Emergency Medicine Residents
Source: MedEdPORTAL. 2026 Jun 17;22:11610. doi: 10.15766/mep_2374-8265.11610 (PMC13272583; doi:10.15766/mep_2374-8265.11610)
Supplement: Supplementary file 1 — Resuscitation Leaders Role.docxTeam and Situational Management.docxResuscitation Guidelines and Psychological Safety.docxResuscitation Leaders Role Review.pptxTeam and Situational Management Review.pptxResuscitation Leadership Escape Room.docxFacilitator Overview Guide.docxLBDQ Form.docxPre- and Postsurvey.docx [file mep_2374-8265.11610-s001.zip › I. Pre- and Postsurvey.docx]

[ ]PGY1 [ ]PGY2 [ ]PGY3

|  | Strongly Disagree | Disagree | Neutral | Agree | Strongly Agree |
| --- | --- | --- | --- | --- | --- |
| I am confident acting as a resuscitation leader |  |  |  |  |  |
| I am confident telling a resuscitation team what is expected of them (through direction and command) |  |  |  |  |  |
| I am confident using uniform guidelines (such as ACLS, PALS) |  |  |  |  |  |
| I am confident maintaining a positive attitude throughout a resuscitation |  |  |  |  |  |
| I am confident deciding WHAT should be done during a resuscitation |  |  |  |  |  |
| I am confident deciding HOW things should be done during a resuscitation |  |  |  |  |  |
| I am confident assigning team members to particular tasks during a resuscitation |  |  |  |  |  |
| I am confident ensuring my role as the resuscitation leader is understood by other team members |  |  |  |  |  |
| I am confident planning the work to be done during resuscitation. |  |  |  |  |  |
| I am confident maintaining a definitive standard (adequate performance) during a resuscitation |  |  |  |  |  |
