# Supplementary material for: An Active-Learning Resuscitation Leadership Curriculum for Emergency Medicine Residents
Source: MedEdPORTAL. 2026 Jun 17;22:11610. doi: 10.15766/mep_2374-8265.11610 (PMC13272583; doi:10.15766/mep_2374-8265.11610)
Supplement: Supplementary file 1 — Resuscitation Leaders Role.docxTeam and Situational Management.docxResuscitation Guidelines and Psychological Safety.docxResuscitation Leaders Role Review.pptxTeam and Situational Management Review.pptxResuscitation Leadership Escape Room.docxFacilitator Overview Guide.docxLBDQ Form.docxPre- and Postsurvey.docx [file mep_2374-8265.11610-s001.zip › _Educational Summary Report_11610.pdf]

MedEdPORTAL
THE AAMC JOURNAL OF TEACHING AND LEARNING RESOURCES

Original Publication

Open Access

An Active-Learning Resuscitation Leadership Curriculum for Emergency Medicine Residents

Michael Sobin, MD*, Peter Prescott, MS, David Berger, MD, Michelle Jankowski, MAS, Danielle Turner-Lawrence, MD, Brett Todd, MD

*Corresponding author: michaelsobin002@gmail.com

Abstract

Introduction: Effective leadership during emergency resuscitations is a core competency for emergency medicine physicians and is associated with improved patient outcomes. Despite its importance, formal training is often limited and informal. A needs assessment at our emergency medicine residency revealed gaps in leadership performance, particularly in communication and role delineation, motivating development of a structured curriculum. **Methods:** We developed a low-cost, active-learning curriculum for emergency medicine residents across all PGYs using Kern's 6-step framework. Educational objectives focused on resuscitation guidelines, role delineation, and team and situational management. The curriculum included 3 flipped-classroom sessions and 1 gamified review, incorporating case-based discussions and role-playing, delivered from July-December 2024. Impact was evaluated with pre- and postimplementation on-shift Leadership Behavior Description Questionnaire assessments and resident confidence surveys. **Results:** Forty-two residents were eligible; 38 (244 assessments) and 33 (136 assessments) residents contributed pre- and postcurriculum bedside assessments, respectively. Leadership performance improved significantly across multiple objectives ($P < .05$), with PGY-1 learners demonstrating the largest gains. Survey responses (30 pre, 28 post) showed significantly increased confidence leading resuscitations across all PGY levels ($P < .001$). Residents valued the interactive and gamified components, highlighting enhanced engagement and exposure to varied leadership strategies. **Discussion:** This low-cost, active-learning curriculum improved emergency medicine residents' resuscitation leadership skills and confidence, particularly among junior learners. Early, structured exposure may foster durable leadership development and enhance team communication. Future work should evaluate the curriculum's longitudinal impact and optimal timing across varied training sites to further refine resuscitation leadership education.

Keywords
Resuscitation, Active Learning, Case-Based Learning, Communication Skills, Emergency Medicine, Flipped Classroom, Games, Leadership Development

Educational Objectives

By the end of this activity, learners will be able to:

1. Communicate clear expectations and roles to members of the resuscitation team during emergency department resuscitations.
2. Direct and coordinate team actions during resuscitations by assigning tasks, prioritizing measures, and guiding how interventions should be performed.
3. Apply standardized resuscitation guidelines to guide team decision-making.
4. Maintain clear performance expectations during the resuscitation.
5. Demonstrate leadership behaviors that promote a composed, supportive, and psychologically safe team environment during the resuscitation.

Citation:
Sobin M, Prescott P, Berger D, Jankowski M, Turner-Lawrence D, Todd B. An active-learning resuscitation leadership curriculum for emergency medicine residents. *MedEdPORTAL*. 2026;22:11610.
https://doi.org/10.15766/mep_2374-8265.11610

Introduction

Effective leadership during resuscitation is a core responsibility of emergency medicine (EM) physicians, requiring rapid execution of life-saving interventions, strong team coordination, decisive clinical judgment, and clear interdisciplinary communication.[1,2] From the beginning of residency, EM trainees are expected to develop these leadership capabilities alongside procedural and diagnostic skills as explicitly outlined in the ACGME EM

Copyright © 2026 Sobin et al. This is an open-access publication distributed under the terms of the Creative Commons Attribution license.

milestones.[3] Strong resuscitation leadership is also associated with improved patient outcomes.[4-6]

Despite its importance, resuscitation leadership remains underemphasized with limited guidance on which specific leadership competencies should be taught.[7,8] Residents often acquire these skills informally through passive observation.[9] Whether traditional training methods adequately prepare graduating EM residents to confidently lead resuscitations remains unclear. Moreover, informal instruction typically lacks defined learning objectives (LOs) and structured assessments, limiting opportunities for feedback and growth.[9,10] At our single-site, tertiary care EM residency program, we conducted a targeted needs assessment of residents' resuscitation leadership skills using the Leadership Behavior Description Questionnaire (LBDQ) during live medical resuscitations.[4] This assessment demonstrated deficits in residents' leadership performance, communication, role delineation, and task assignment. These findings informed the development of a structured curriculum targeting key resuscitation leadership knowledge, skills, and behaviors.

Few published curricula have addressed resuscitation leadership training specifically for EM residents. Gartland et al.[11] employed simulation-based learning to improve crisis-resource management skills. While simulation is an effective method for teaching procedural and behavioral competencies, it can be limited by high cost and time requirements.[12] Hegarty et al.[13] used traditional didactic strategies to convey resuscitation leadership competencies. However, the traditional lecture format is less effective for teaching skills and attitudes required for resuscitation leadership competency.[12] Recognizing these challenges, our team aimed to develop a cost-effective, active-learning curriculum to teach essential resuscitation leadership knowledge, skills, and behaviors.

Active-learning strategies have been applied in EM education with evidence of positive impacts on EM learner engagement, knowledge acquisition, and contextualization of learned material.[14-16] Gamified didactics, educational activities that incorporate game design principles, have also proven effective in fostering higher levels of learner engagement and motivation.[17] Building on the utility of these active, low-cost strategies, we developed a novel, low-fidelity resuscitation leadership curriculum for EM residents. This curriculum integrates flipped-classroom strategies, role-play case-based discussions, and gamification to provide comprehensive, accessible training in resuscitation leadership. The purpose of this article is to describe the development, implementation, and evaluation of this curriculum, which we hypothesized would enhance EM residents' confidence and competence in resuscitation leadership.

Methods

The target audience for this curriculum was EM residents at all levels of training. We designed the curriculum to be facilitated by attending physicians or senior residents with additional resuscitation or leadership experience or training, such as chief residents who undergo additional leadership instruction in preparation for their roles. Facilitators had prior experience delivering flipped-classroom–style didactics, as faculty and senior residents at our institution regularly delivered Foundations of Emergency Medicine flipped-classroom cases. Learners were expected to have baseline knowledge of Advanced Cardiovascular Life Support/Pediatric Advanced Life Support resuscitation protocols.

Curriculum development followed Kern's 6-step approach,[12] a validated and widely applied framework for curriculum development in health professions education. A general and targeted needs assessment identified gaps in resuscitation leadership training both in the literature and at our institution. Following general and targeted needs assessments, the authors created a resuscitation leadership curriculum aimed at strengthening communication and team management skills within emergency department resuscitations. Drawing on author expertise and a review of the resuscitation leadership literature, we used the LBDQ to shape the curriculum's LOs.[4] The LBDQ, a validated leadership assessment tool of in-hospital resuscitations, has been employed in multiple prior resuscitation leadership studies and educational interventions.[5,18]

Given resource limitations identified during curriculum planning meetings and the scarcity of published low-resource interventions for resuscitation leadership education, the authors selected active-learning strategies that required minimal technology. Specifically, we incorporated flipped-classroom sessions, case-based discussions, role-playing, and escape rooms, drawing on our department's experiences with similar strategies.

Curriculum content was developed through a review of key literature on resuscitation leadership theory, existing curricular models, and other medical leadership instructional approaches. We selected self-directed learning materials based on relevance to the LOs and clarity for EM residents. Optional free open-access medical education resources were provided for learners interested in exploring topics beyond the required material. The authors disseminated the preparatory materials to learners

Copyright © 2026 Sobin et al. This is an open-access publication distributed under the terms of the Creative Commons Attribution license.

2 weeks before each didactic session, with a reminder sent 1 week prior. Review of the preparatory materials for session one (Appendix A), session two (Appendix B), and session three (Appendix C) was expected to take learners approximately 30 minutes.

The curriculum consisted of 3 flipped-classroom sessions and 1 gamified review session, each tied to subsets of the LBDQ-based LOs: (1) defining the resuscitation leader's role (see Appendix A, LO 1), (2) team and situational management (see Appendix B, LO 2), and (3) applying resuscitation guidelines and promoting psychological safety (see Appendix C, LOs 3-5). The first 2 didactic sessions were preceded by a brief PowerPoint review of the preassigned learning materials (Appendices D and E). The 3 flipped-classroom sessions used role-playing and case-based discussions to review the learning material. We delivered the final review session as a case-based escape room that reinforced concepts from prior sessions (Appendix F). Each session lasted approximately 30-60 minutes.

Facilitators reviewed discussion guides in advance, which required 15-30 minutes of preparation. Facilitators were encouraged to review the associated prereading materials; however, this was not required, as key concepts were summarized within the session guides. A facilitator instructional guide was developed to outline facilitator expectations, flipped-classroom facilitation strategies, and challenging learner approaches (Appendix G).

Necessary materials and space for each didactic included a room that could be easily configured for group activities, copies of the role-playing or escape room documents in each session's appendix, and audiovisual equipment for a brief presentation of the preassigned learning materials.

Curriculum implementation occurred over the first 6 months of the academic year in the fall of 2024, with didactic sessions offered every 6 weeks during EM conference hours, totaling 4 hours of conference time. The curriculum was integrated into the existing resident conference schedule and spaced with sessions 4-6 weeks apart to promote spaced repetition and reinforce longitudinal learning. Residents were required to attend unless assigned to clinical duties, on vacation, or ill. To ensure broad access, all learning materials, including discussion guides and handouts, were shared electronically.

We evaluated the curriculum's impact on residents' confidence and leadership performance using both subjective and objective pre- and postimplementation assessments. All EM residents in good standing ($N = 42$) were eligible to participate. One year prior to curriculum implementation, the authors conducted bedside assessments of EM residents during medical resuscitations, defined as nontraumatic cases requiring immediate EM physician intervention, using the LBDQ for leadership assessment. In our department, such cases are assigned to a high-acuity bed, and the resident and supervising attending meet the patient at the bedside. Residents at all training levels respond to and lead resuscitations under faculty supervision. Faculty assessed the resident's leadership performance during these encounters using a modified LBDQ (Appendix H) with a 3-point scale (performed, partially performed, not performed) for assessment efficiency and accuracy based on pilot feedback. Faculty received electronic instructions via email on how to complete the form, which were reviewed at monthly faculty meetings. The authors piloted the process prior to the initial pretest in December 2023 through trial bedside assessments with faculty while study authors were present.

Aggregated forms represent a convenience sample. For each case, the encounter date and resident PGY were recorded. Additional resident identifiers were removed during data abstraction to ensure anonymity. Exclusion criteria included cases involving trauma or incomplete observations. Trauma cases were specifically excluded because other consultant teams shared leadership responsibilities. We conducted pretest assessments from January 2024 to June 2024 and posttest assessments from January 2025 to March 2025, corresponding to the same portion of the academic year. Because residents advanced in PGY during this period, the pre- and postassessment groups represented a partially overlapping cohort. Due to scheduling limitations among the authors performing data collection and analysis, the postimplementation assessment period was shorter than the preimplementation assessment period. This evaluation aligned with Kirkpatrick's third level (behavior).[19] The Institutional Review Board at Corewell Health William Beaumont University Hospital deemed this study non-human subject research (2023-223). For the bedside LBDQ assessments, each completed form was treated as an independent observation. Scores for each LO were analyzed separately. Pre- and postcurriculum mean LO scores were compared using 2-sample *t* tests. Analyses were unadjusted, and no correction was made for multiple comparisons. PGY subgroups were evaluated using the same univariate methods. All statistical analyses were conducted using SAS version 9.4 (SAS Institute, Cary, NC). Statistical significance was defined as $P < .05$.

We also measured resident comfort with resuscitation leadership skills and individual LOs using pre- and postcurriculum surveys

Copyright © 2026 Sobin et al. This is an open-access publication distributed under the terms of the Creative Commons Attribution license.

(Appendix I). The authors developed the survey to allow residents to rate their comfort acting as a resuscitation leader and performing the LBDQ-derived LOs on a 5-point Likert scale (1: *strongly disagree*, 2: *disagree*, 3: *neutral*, 4: *agree*, 5: *strongly agree*). The precurriculum survey was completed in July 2024, immediately prior to the initial didactic. Immediately following the final review, residents completed a postcurriculum survey in December 2024 identical to the precurriculum survey. These surveys addressed Kirkpatrick's first level (reaction).[19] Resident identity was kept anonymous by removing identifying information during data collection. Pre- and postcurriculum survey responses were treated as independent (unmatched) samples. Each Likert-scale item, including overall leadership confidence and individual LBDQ-derived LOs, was analyzed separately. Mean pre- and postcurriculum scores were compared using 2-sample *t* tests. Analyses were unadjusted, and no correction was applied for multiple comparisons. Statistical significance was defined as $P < .05$. All analyses were performed using SAS version 9.4 (SAS Institute, Cary, NC).

Lastly, resident perceptions of the curriculum were assessed through written feedback. At the end of each didactic session, residents were given a standard evaluation form and invited to provide voluntary, anonymous written commentary, suggestions, and feedback. Narrative responses were reviewed at the conclusion of the curriculum using a simple thematic analysis approach. The study team collectively reviewed the comments to identify recurring themes related to the curriculum's strengths and opportunities for improvement.

Results

All residents attended at least 1 didactic session. An average of 24 of 42 residents (57.1%) participated per session, with attendance of 22 residents for session one, 24 in session two, 21 in session three, and 28 in session four. All residents had asynchronous access to all learning materials. For the bedside LBDQ assessments, we completed 244 forms (average 5.8, range 0-17) on 38 of 42 residents (90.5%) during the pretest period in the spring of 2024 (12 PGY1, 12 PGY2, 14 PGY3) and 136 forms (average 3.2, range 0-13) on 33 of 42 residents (78.6%) during the posttest period in the spring of 2025 (9 PGY1, 10 PGY2, 14 PGY3). Overall, resident performance improved across most LOs (Figure 1), with statistically significant improvements on LO 1 (let a team know what is expected of them, $P = .012$), LO 2 (demonstrate the use of uniform guidelines, $P = .027$), LO 5 (decide how things should be done, $P = .011$), LO 6 (assign group members to particular tasks, $P < .001$), LO 7 (make sure their part in the team was understood by team members,

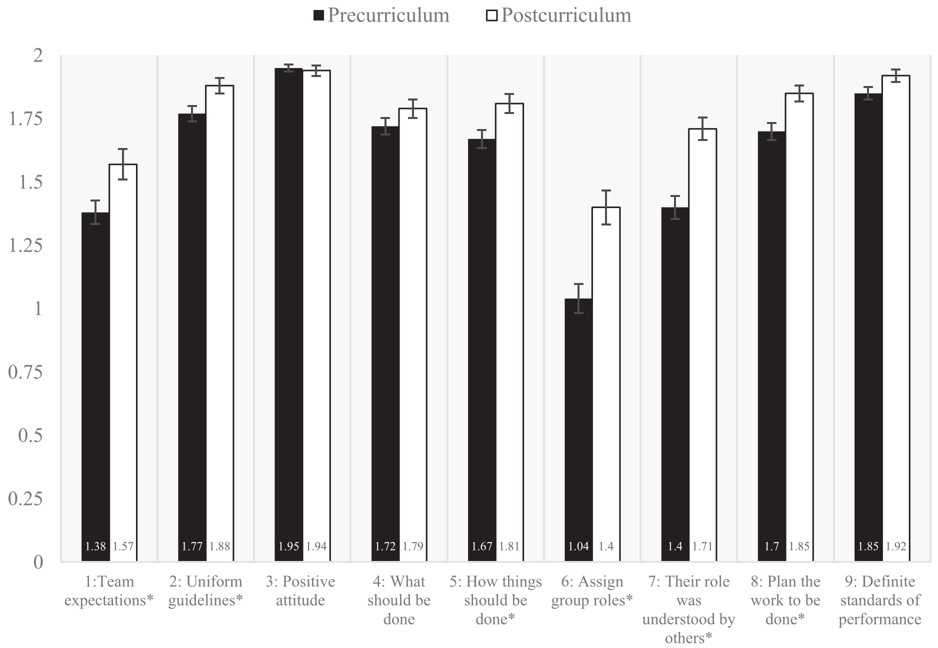

Figure 1. Average overall resident LBDQ bedside leadership assessment scores with standard error (error bars) for the pre- ($N = 38$) and postcurriculum ($N = 33$) assessment periods, measured on a 3-point scale (0 = *not performed*, 1 = *partially performed*, 2 = *performed*). Abbreviation: LBDQ, Leadership Behavior Description Questionnaire. * $P < .05$.

Copyright © 2026 Sobin et al. This is an open-access publication distributed under the terms of the Creative Commons Attribution license.

$P < .001$), and LO 8 (plan the work to be done, $P = .004$). PGY1 learners exhibited the largest improvement in leadership performance, with statistically significant gains across 7 LOs (Figure 2). Notably, LO 7 (make sure their part in the team was understood by team members) improved significantly across all PGY classes (see Figure 2, $P < .05$).

For the leadership confidence surveys, 30 of 42 residents (71.4%) completed the precurriculum survey (14 PGY1, 8 PGY2, 8 PGY3), and 28 of 42 residents (66.7%) completed the postcurriculum survey (9 PGY1, 9 PGY2, 10 PGY3). Resident-reported confidence acting as a resuscitation leader improved significantly after the curriculum ($P < .001$). PGY3 confidence acting as the resuscitation leader did not change significantly after the curriculum. Additionally, confidence performing each LBDQ LO also improved significantly ($P < .001$) after the curriculum (Figure 3), with consistent improvement across all PGY classes (Figure 4, $P < .05$) except for LO 3 (display a positive attitude).

Residents reacted positively to the curriculum overall in their written feedback. Positive feedback included appreciation for the interactive nature of the case-based discussions and gamified components, as well as insights into various leadership approaches they had not previously encountered. Suggested areas for improvement included unclear roles during case-based scenarios and ensuring more junior learners had equal access to leadership opportunities.

Discussion

This resuscitation leadership curriculum, developed using Kern's 6 steps, aimed to enhance EM residents' confidence, knowledge, and skills in leading resuscitations through student-centered, active-learning strategies. Following participation, residents reported increased confidence leading resuscitations and demonstrated measurable improvement in leadership knowledge and observable team leadership behaviors.

Notably, the greatest gains were observed among junior residents, suggesting that early structured leadership training enhanced longitudinal leadership development. This finding underscores the value of introducing leadership concepts early, even before learners are expected to independently lead the entirety of a resuscitation. Prior studies in undergraduate medical education demonstrate that early leadership curricula can enhance learners' confidence and competence.[20-22] Furthermore, early acquisition of foundational leadership behaviors may foster more longitudinal leadership development, consistent with other longitudinal educational interventions.[23,24] However, few studies have examined the longitudinal impact of early

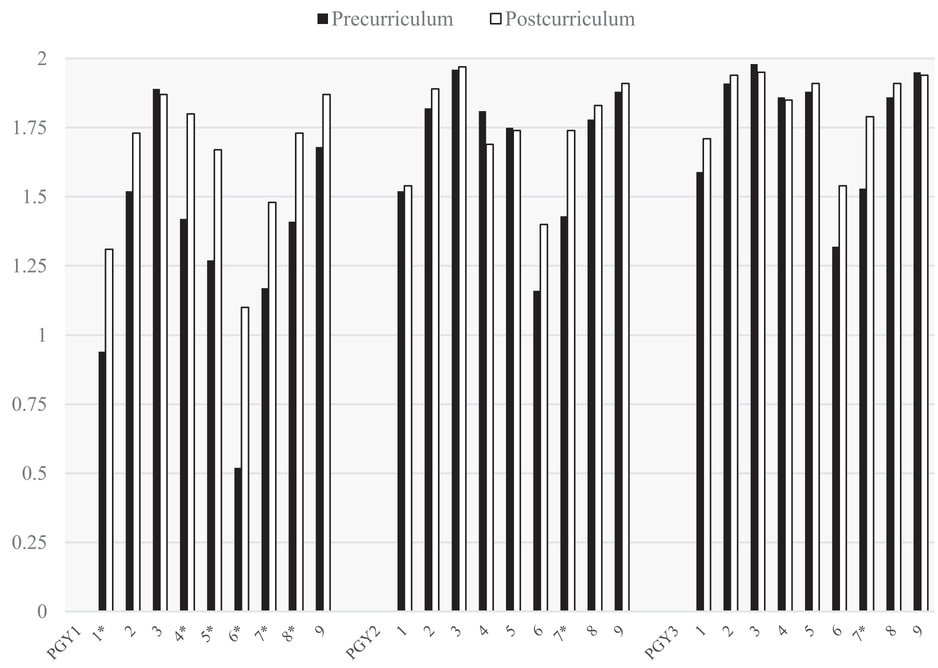

Figure 2. Average resident LBDQ bedside leadership assessment scores per PGY for the pre- (12 PGY1, 12 PGY2, 14 PGY3) and postcurriculum (9 PGY1, 10 PGY2, 14 PGY3) assessment periods, measured on a 3-point scale (0 = *not performed*, 1 = *partially performed*, 2 = *performed*). Abbreviation: LBDQ, Leadership Behavior Description Questionnaire. * $P < .05$.

Copyright © 2026 Sobin et al. This is an open-access publication distributed under the terms of the Creative Commons Attribution license.

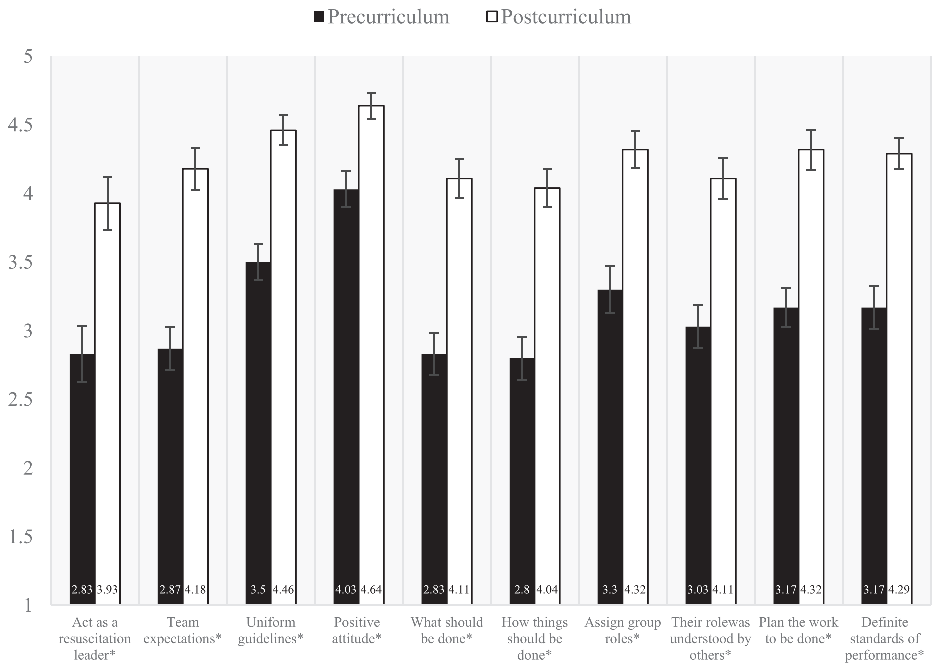

Figure 3. Average resident leadership confidence with standard error (error bars) for the pre- ($N = 30$) and postcurriculum ($N = 28$) surveys, measured on a 5-point Likert scale (1 = *strongly disagree*, 2 = *disagree*, 3 = *neutral*, 4 = *agree*, 5 = *strongly agree*). Abbreviation: LBDQ, Leadership Behavior Description Questionnaire. * $P < .05$.

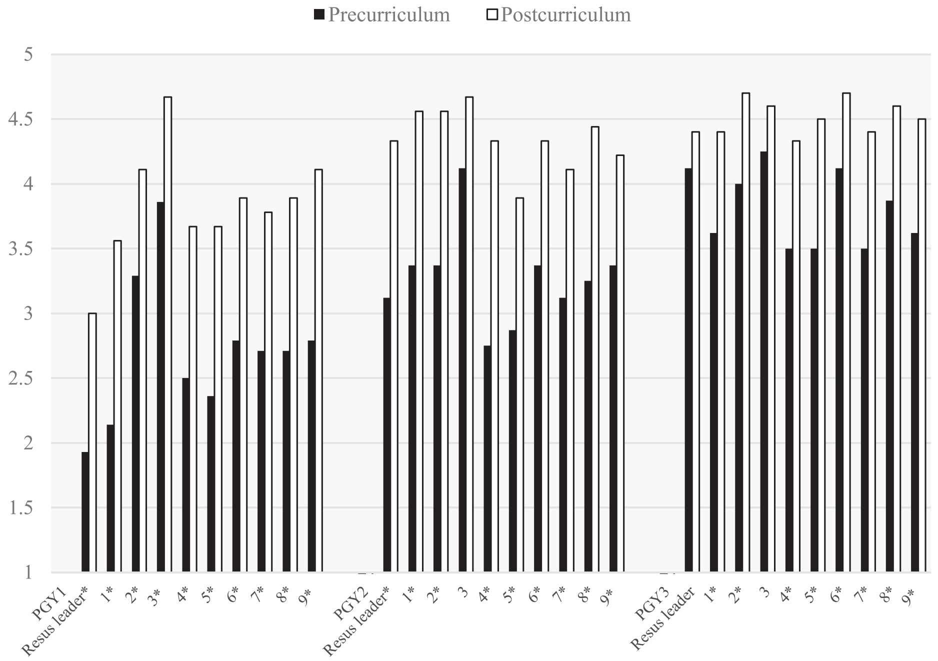

Figure 4. Average resident leadership confidence per PGY for the pre- (14 PGY1, 8 PGY2, 8 PGY3) and postcurriculum (9 PGY1, 9 PGY2, 10 PGY3) ($N = 9$) surveys, measured on a 5-point Likert scale (1 = *strongly disagree*, 2 = *disagree*, 3 = *neutral*, 4 = *agree*, 5 = *strongly agree*). Abbreviation: LBDQ, Leadership Behavior Description Questionnaire. * $P < .05$.

Copyright © 2026 Sobin et al. This is an open-access publication distributed under the terms of the Creative Commons Attribution license.

leadership education, and none have explored its effects on resuscitation leadership in EM. While our findings suggest that implementation early in residency may be particularly advantageous, future work should explore the optimal timing and frequency of resuscitation leadership instruction. Local factors, such as faculty availability and program resources, should inform the timing of implementation. Prior curricula have used varied approaches, including didactic and longitudinal models,[2,25] although brief, longitudinal reinforcement with periodic simulation-based refreshers may be more effective than a single isolated intervention.

Improvements in team communication skills were among the most notable outcomes of our curriculum across all training levels. Prior to implementation, residents demonstrated challenges in role assignment and clarifying leadership responsibilities, with team communication objectives scoring lowest among all assessed competencies. We hypothesize that this may relate to our high-acuity clinical setting, where frequent exposure to critically ill patients and highly experienced nursing may lessen the need for residents to take a directive leadership role compared to a more facilitative, empowering leadership role.[26] However, because the majority of emergency physicians ultimately practice in smaller or community-based hospitals, where resuscitation team members may have variable experience and training, developing directive leadership skills and clear communication behaviors during residency is essential.[27] Prior studies have emphasized that effective leadership in high-acuity, low-resource environments relies heavily on explicit communication and shared mental modeling.[26] As residency programs align with ACGME expectations for training in diverse and resource-limited settings, introducing these skills early may help residents adapt to a wide range of clinical environments. That our curriculum improved team communication across all PGYs suggests it may be a valuable tool to prepare residents for effective communication during emergency resuscitations in variable resource settings.

Several design choices appeared to enhance the learning experience for residents. Facilitator observations suggested that a mixed-level small group fostered a collaborative learning environment, allowing junior residents to benefit from the perspectives and experiential insights of senior peers. This structure may promote informal mentorship and peer-to-peer learning, yet these outcomes were not systematically assessed in this study. Although objective assessments demonstrated smaller gains for senior residents, we believe that participation reinforced their leadership and mentorship skills, competencies that are critical for both clinical practice and future educational roles.[3] This curriculum integrated small-group learning, flipped-classroom methods, and gamification principles, which are well established in health professions education as effective tools for enhancing engagement and retention of medical knowledge.[16,17,28] Resident feedback suggested that this interactive format was well received and engaging, potentially supporting sustained participation. Our findings also suggest that active-learning strategies may contribute to measurable improvements in leadership performance. Future work comparing active-learning approaches with traditional lecture-based formats in leadership training may help clarify the impact of different instructional strategies. Furthermore, future research should explore whether these approaches can similarly promote skill development in related domains such as time management, situational awareness, and human factors training. It should be noted that prior to rollout, our residents were already familiar with team-based and active-learning strategies, which likely facilitated a smoother adoption of this curriculum. Introducing this curriculum in other settings may similarly benefit from prior exposure to these educational methods within their didactics to optimize the likelihood of success.

Our curriculum has several limitations. The generalizability of our findings is limited by the single-site design of the curriculum assessment, as institutional culture and resident baseline characteristics may differ across EM programs. Implementation and evaluation at additional training sites will be necessary to validate our results. Because not all residents in our program attended all didactic sessions, incomplete exposure may have affected outcomes. To mitigate this, all materials were made available for residents to review independently after each session. Engagement with prereading and optional learning materials was not tracked, limiting insight into learner engagement and the impact of flipped-classroom preparation on observed outcomes. Lastly, the confidence survey response rates of 71% precurriculum and 67% postcurriculum introduce the potential for nonresponse bias.

Our assessment approach also introduced potential sources of bias. The use of convenience sampling in an uncontrolled clinical environment may limit the validity of our results. Additionally, because leadership confidence was assessed longitudinally during residency, maturation bias remains a consideration. There was an uneven distribution of presurvey responses across PGY classes, which may have introduced nonresponse bias into the

Copyright © 2026 Sobin et al. This is an open-access publication distributed under the terms of the Creative Commons Attribution license.

overall presurvey results. However, results were also analyzed and presented by PGY class to help mitigate this limitation. Survey and observational assessment data were also collected anonymously to protect resident confidentiality. As a result, individual attendance at specific curriculum sessions could not be linked to survey or assessment outcomes. This prevented analysis of the relationship between in-person curriculum attendance and improvements in leadership performance and confidence. Additionally, we did not use a formal control group in our study design. We attempted to mitigate these limitations by using a pre–post design for the objective assessment conducted during the same portion of the academic year, with preintervention performance serving as an internal comparator to reduce the influence of external learning factors and maturation for the objective assessment. Next, the postimplementation assessment period was shorter than the preimplementation period, resulting in fewer completed on-shift assessments. This unequal observation window may have limited opportunities to capture resident leadership behaviors and could introduce sampling variability between the pre- and postcurriculum groups. Finally, although we adapted a previously validated leadership assessment instrument,[4] for ease of use the rating scale was modified from a 5- to 3-point Likert scale without additional validation. This change may have impacted scale sensitivity.

We are eager to explore several opportunities to expand this curriculum, including incorporating additional assessment tools, such as video review of resident-led resuscitations, to better understand resident leadership development. We are also interested in exploring the curriculum's longitudinal effects, particularly how timing within residency training influences outcomes.

In conclusion, this curriculum offers a low-cost, active-learning approach to developing resuscitation leadership knowledge and skills among EM residents. It provides a replicable model for programs seeking to prepare residents to lead resuscitations effectively during training and in independent practice through engaging, evidence-informed educational strategies.

Appendices

A. Resuscitation Leaders Role.docx
B. Team and Situational Management.docx
C. Resuscitation Guidelines and Psychological Safety.docx
D. Resuscitation Leaders Role Review.pptx
E. Team and Situational Management Review.pptx
F. Resuscitation Leadership Escape Room.docx
G. Facilitator Overview Guide.docx
H. LBDQ Form.docx
I. Pre- and Postsurvey.docx

All appendices are peer reviewed as integral parts of the Original Publication.

Michael Sobin, MD: Assistant Professor, Department of Emergency Medicine, Medical College of Wisconsin; ORCID: https://orcid.org/0000-0003-1595-8265

Peter Prescott, MS: Medical Student, Oakland University William Beaumont School of Medicine

David Berger, MD: Associate Professor, Department of Emergency Medicine, Corewell Health William Beaumont University Hospital

Michelle Jankowski, MAS: Biostatistician, Oakland University William Beaumont School of Medicine

Danielle Turner-Lawrence, MD: Associate Professor, Department of Emergency Medicine, Corewell Health William Beaumont University Hospital

Brett Todd, MD: Associate Professor, Department of Emergency Medicine, Corewell Health William Beaumont University Hospital

Disclosures
None to report.

Funding/Support
None to report.

Ethical Approval
Corewell Health William Beaumont University Hospital Institutional Review Board reviewed this project.

References

1. Lateef F. Grace under pressure: leadership in emergency medicine. *J Emerg Trauma Shock*. 2018;11(2):73-79. https://doi.org/10.4103/JETS.JETS_18_18
2. Zdradzinski MJ, Sanders S, Kazmi Q, Fields V, O'Shea J, Medoff S. A novel leadership curriculum for emergency medicine residents. *J Educ Teach Emerg Med*. 2024;9(1):C1-C15. https://doi.org/10.21980/j81d2s

Copyright © 2026 Sobin et al. This is an open-access publication distributed under the terms of the Creative Commons Attribution license.

3. Beeson MS, Carter WA, Christopher TA, et al. The development of the emergency medicine milestones. *Acad Emerg Med*. 2013;20(7):724-729. https://doi.org/10.1111/acem.12157
4. Cooper S, Wakelam A. Leadership of resuscitation teams: "Lighthouse Leadership." *Resuscitation*. 1999;42(1):27-45. https://doi.org/10.1016/S0300-9572(99)00080-5
5. Yeung JHY, Ong GJ, Davies RP, Gao F, Perkins GD. Factors affecting team leadership skills and their relationship with quality of cardiopulmonary resuscitation. *Crit Care Med*. 2012;40(9): 2617-2621. https://doi.org/10.1097/CCM.0b013e3182591fda
6. Chan JL, Lehrich J, Nallamothu BK, et al; American Heart Association's Get With the Guidelines®-Resuscitation Investigators. Association between hospital resuscitation champion and survival for in-hospital cardiac arrest. *J Am Heart Assoc*. 2021;10(5):e017509. https://doi.org/10.1161/JAHA.120.017509
7. Rosenman ED, Branzetti JB, Fernandez R. Assessing team leadership in emergency medicine: the milestones and beyond. *J Grad Med Educ*. 2016;8(3):332-340. https://doi.org/10.4300/JGME-D-15-00400.1
8. Sobin M, Todd B, Chen NW, Turner-Lawrence D. Resuscitation leadership education: a needs assessment of emergency medicine residencies. *West J Emerg Med*. 2025;27(1):33-38. https://doi.org/10.5811/WESTJEM.47285
9. Goldman EF, Plack MM, Roche CN, Smith JP, Turley CL. Learning clinical versus leadership competencies in the emergency department: strategies, challenges, and supports of emergency medicine residents. *J Grad Med Educ*. 2011;3(3):320-325. https://doi.org/10.4300/JGME-D-10-00193.1
10. Lawrence C, Mhlaba T, Stewart KA, Moletsane R, Gaede B, Moshabela M. The hidden curricula of medical education: a scoping review. *Acad Med*. 2018;93(4):648-656. https://doi.org/10.1097/ACM.0000000000002004
11. Gartland R, Conlon L, Livingston S, Glick JE, Bach G, Abboud ME. Resuscitation leadership training: a simulation curriculum for emergency medicine residents. *MedEdPORTAL*. 2022;18:11278. https://doi.org/10.15766/mep_2374-8265.11278
12. Thomas PA, Kern DE, Hughes MT, Chen BY, eds. *Curriculum Development for Medical Education: A Six-Step Approach*. Johns Hopkins University Press; 2016.
13. Hegarty C, Barringer K, Nelson J, Raghunandan S, Binstadt E. ABC's of team leadership in emergency medicine. *MedEdPORTAL*. 2013;9:9552. https://doi.org/10.15766/mep_2374-8265.9552
14. Wolff M, Wagner MJ, Poznanski S, Schiller J, Santen S. Not another boring lecture: engaging learners with active learning techniques. *J Emerg Med*. 2015;48(1):85-93. https://doi.org/10.1016/j.jemermed.2014.09.010
15. Lew EK. Creating a contemporary clerkship curriculum: the flipped classroom model in emergency medicine. *Int J Emerg Med*. 2016;9(1):25. https://doi.org/10.1186/s12245-016-0123-6
16. Moore KG, Ketterer A, Wheaton N, et al. Development, implementation, and evaluation of an open access, level-specific, core content curriculum for emergency medicine residents. *J Grad Med Educ*. 2021;13(5):699-710. https://doi.org/10.4300/JGME-D-21-00067.1
17. Gue S, Ray J, Ganti L. Gamification of graduate medical education in an emergency medicine residency program. *Int J Emerg Med*. 2022;15(1):41. https://doi.org/10.1186/s12245-022-00445-1
18. Rosenman ED, Ilgen JS, Shandro JR, Harper AL, Fernandez R. A systematic review of tools used to assess team leadership in health care action teams. *Acad Med*. 2015;90(10):1408-1422. https://doi.org/10.1097/ACM.0000000000000848
19. Robinson D, Chan TM, Krzyzaniak SM, et al., eds. *Education Theory Made Practical*, vol. 3. Academic Life in Emergency Medicine; 2020.
20. Richard K, Noujaim M, Thorndyke LE, Fischer MA. Preparing medical students to be physician leaders: a leadership training program for students designed and led by students. *MedEdPORTAL*. 2019;15:10863. https://doi.org/10.15766/mep_2374-8265.10863
21. Nicolaou N, Nicolaou C, Nicolaou P, Nicolaides P, Papageorgiou A. Development of a leadership and management module for the undergraduate medical curriculum. *BMC Med Educ*. 2024;24(1):1310. https://doi.org/10.1186/s12909-024-06004-x
22. Lisevick A, Dreiling J, Chauvin S, et al. Leadership for medical students: lessons learned from a relationship-centered leadership curriculum. *Am J Lifestyle Med*. 2022;17(1):56-63. https://doi.org/10.1177/15598276221106940
23. Steinbach TC, Adamson R, Carlbom DJ, et al. Crisis leadership education for critical care fellows. A longitudinal curriculum using simulation. *ATS Sch*. 2020;1(1):11-19. https://doi.org/10.34197/ats-scholar.2019-0005IN
24. Bang S, Bahl A. Impact of early educational intervention on coding for first-year emergency medicine residents. *AEM Educ Train*. 2018;2(3):213-220. https://doi.org/10.1002/aet2.10102
25. Jaramillo-Restrepo V, Losee JE, Bump GM, Bison-Huckaby M, Merriam S. Educating our future medical leaders: an innovative longitudinal course across surgical and medical specialties in graduate education. *J Healthc Leadersh*. 2024;16:255-262. https://doi.org/10.2147/JHL.S468061
26. Yun S, Faraj S, Xiao Y, Sims HP. Team leadership and coordination in trauma resuscitation. In: Beyerlein M, Johnson D, Beyerlein S, eds. *Team-Based Organizing: Advances in Interdisciplinary Studies of Work Teams*. Vol. 9. Emerald Group Publishing Limited; 2003:189-214. https://doi.org/10.1016/S1572-0977(02)09011-8

Copyright © 2026 Sobin et al. This is an open-access publication distributed under the terms of the Creative Commons Attribution license.

27. Ford K, Menchine M, Burner E, et al. Leadership and teamwork in trauma and resuscitation. *West J Emerg Med*. 2016;17(5): 549-556. https://doi.org/10.5811/westjem.2016.7.29812

28. DiCesare D, Scheveck B, Adams J, et al. The pit crew card game: a novel gamification exercise to improve EMS performance in critical care scenarios. *Int J Emerg Med*. 2025;18(1):25. https://doi.org/10.1186/s12245-024-00748-5

Received: December 12, 2025
Accepted: May 8, 2025
Published: June 17, 2026

Copyright © 2026 Sobin et al. This is an open-access publication distributed under the terms of the Creative Commons Attribution license.
